# Examining the research methods of early warning signals in clinical psychology through a theoretical lens

**DOI:** 10.1186/s12888-025-06688-5

**Published:** 2025-03-19

**Authors:** Jingmeng Cui, Merlijn Olthof, Fred Hasselman, Anna Lichtwarck-Aschoff

**Affiliations:** 1 Faculty of Behavioural and Social Sciences, University of Groningen, Grote Kruisstraat 2/1, Groningen, 9712 TS The Netherlands; 2 Behavioural Science Institute, Radboud University, Nijmegen, The Netherlands

**Keywords:** Early warning signals, Critical transitions, Sudden changes, Complex dynamical systems

## Abstract

**Background:**

The past few years have seen a rapid growth in research on early warning signals (EWSs) in the psychopathology domain. Whereas early studies found EWSs to be associated with sudden changes in clinical change trajectories, later findings showed that EWSs may not be general across variables and cases and have low predictive power. These mixed results may be explained by the diverse methods employed in clinical EWS studies, with some of these approaches and practices potentially misaligned with the underlying theory of EWSs.

**Methods:**

This article employs a variety of methods, such as a narrative review, mathematical derivations, simulations, and visual illustrations, to support our claims, explain specific assumptions, and guide future empirical research. This multitude of methods serves our aim to provide theoretical as well as methodological contributions to the field.

**Results:**

We identify the following key assumptions for EWS validation studies: the system departs from a point attractor, EWSs appear before the critical transition, and EWS variables align with system destabilization. The literature review shows that the common research practices in the field are often not in line with those assumptions, and we provide specific suggestions corresponding to each of the assumptions.

**Conclusions:**

More rigorous empirical evidence is needed to better validate the existence of EWSs in clinical sudden changes and fully realize their clinical potential. As theory-based prediction tools, EWSs require stronger alignment between theory and practice to enhance both theoretical understanding and predictive accuracy.

**Clinical trial number:**

Not applicable.

**Supplementary Information:**

The online version contains supplementary material available at 10.1186/s12888-025-06688-5.

## Background

Clinical change is not always linear and gradual. In fact, about half of the clients undergo one or more sudden changes in their symptom trajectory over the course of treatment [1–3]. Those sudden changes are difficult to predict, yet have important clinical implications because they are often closely linked to treatment outcomes [1, 3]. Recently, researchers have proposed to use early warning signals (EWSs) to predict sudden changes in clinical trajectories [4–7]. EWSs are a group of statistical indicators depicting the instability of a complex system, that are generally observable before a system changes to a new stable state. Intuitively, the existence of EWSs can be understood by the following idea. Different *phases*^1^ may emerge from the interactions among interdependent biological, psychological, and sociocultural factors within a complex person-environment system. In psychopathology, those phases may represent mental suffering (e.g. depressed mood, anxiety, panic), but also a healthy, well-functioning phase [8, 10–14]. Often, such phases are relatively stable, which means that the system may be stuck in a certain phase, having difficulties disengaging or escaping from it. For example, people with depression may be stuck in a phase characterized by depressed mood, reduced sleep quality, and difficulties in concentration [12, 15]. Yet the stability of phases may decrease over time, making it easier for the system to escape. The destabilization of the unhealthy phase, which corresponds to the loosening of undesirable cognitive, affective, or behavioral patterns [12], then functions as a mechanism for the sudden gain toward a healthier phase. Likewise, the destabilization of a healthy phase may function as a mechanism for the sudden loss toward the unhealthy phase. When a certain phase is destabilized, the system state is more likely to fluctuate, and after perturbation, the system takes longer to recover. As a result, various early-warning indicators, such as increasing variance (associated with stronger fluctuations) and autoregressive coefficients (associated with slower recovery), can be observed in the data.^2^ Therefore, we may use the EWSs evidenced in measurements of the mental state of a client to predict whether the client is likely to have a sudden change soon.

Several studies have found empirical evidence that EWSs may exist for clinical changes [19–21] and hypothesized that EWSs can potentially be used for detecting vulnerable individuals, determining the timing of interventions, and predicting the direction of change [22, 23]. Later studies, however, found the predictive power of EWSs to be generally weak, EWSs only occurring in some variables different for each client, and overall, not showing a clear consistent pattern [24–28]. Those mixed findings may arise from individual differences or measurement techniques, but it is also likely that they stem from methodological issues [29]. Considering the relative novelty of the field, various research methodologies and paradigms to study EWS coexist. Some studies monitor symptom levels with frequently repeated measures [19, 20], and some studies only use pre-and post-assessments to evaluate whether a change has occurred [25, 30]; some studies use emotion items to calculate EWSs [20, 24, 25], and others use treatment process measures, for example, therapy progress and insight [19]. Among this variety of methods, some might be more suitable than others, yielding more reliable results. Assessing these methods requires a thoughtful examination of their alignment with the foundational theory of EWSs. While some studies and reviews have discussed several potential issues of research methods in this field [28, 29], we identified several important facets that have received limited attention. To address these points, a rigorous evaluation is needed to establish a direct link between the mathematical theory of EWSs and specific research practices in psychopathology. Therefore, in this paper, we aim to formulate recommendations for improved research methodology in clinical EWS studies based on a mathematical derivation of EWSs in multivariate dynamic systems.

## Methods

The current article is a theoretical and methodological contribution. We apply multiple methods to support our claims, evaluate the common research practice in the field, and provide practical suggestions for empirical researchers.

We first provide a mathematical, theoretical derivation of EWSs in general multivariate dynamic systems, which clarifies the original EWS theory that psychopathology borrowed from complexity science. We show the mathematical derivations in Supplementary Materials A, and in the main text, we explain the gist of the derivations with verbal language, visual illustrations, and simulations (with the simulation details in Supplementary Materials B). From the derivation, we identify three key assumptions that should be considered when designing EWS studies: the system departs from a point attractor, EWSs appear before the critical transition, and EWS variables align with system destabilization.

After that, we elaborate on each assumption and discuss their implications for research in the psychopathology domain. For each assumption, we investigate whether the research practices in previous empirical studies were in line with those assumptions and provide suggestions for future studies. We used narrative reviews considering the field of EWS studies in psychopathology is still in its initial stage and the number of empirical studies is rather limited. Some other methods were also used to better explain each specific assumption. For the first assumption, we introduce a new visual check method, namely the distance plot, to assist researchers in examining if this assumption is met. We also include brief simulations and empirical examples for this method. For the third assumption, we provide a brief literature review of the EWS investigations in other scientific fields to illustrate the difference in research methods between psychopathology and other disciplines.

Finally, we provide an integrative summary and discussion of our findings.

## Results

### The formal theory of EWSs

**Fig. 1:**
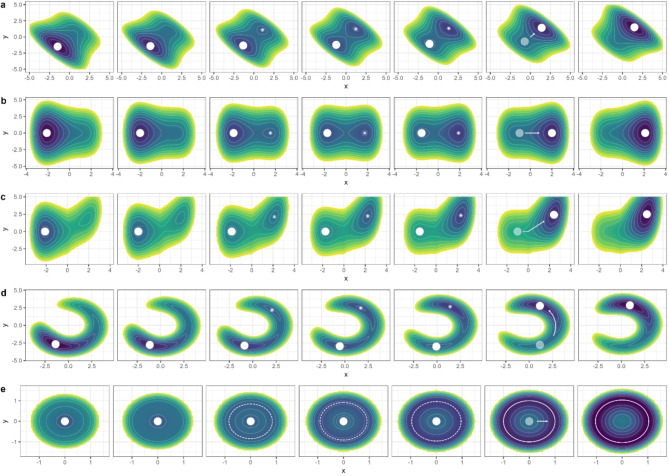
Different examples for bifurcation-induced transitions. Colors that are darker blue represent deeper regions of the stability landscape, and therefore stabler system states. Points and solid circles represent the actual state of the system, and asterisks and dashed circles represent the attractors that are not occupied by the system. For each example, the tipping point is at the second column from the right, represented by arrows. (**a**) The direction of stability loss and the transition involve both the *x*- and the *y*-axes. (**b**) The direction of stability loss and the transition involve only the *x*-axis. (**c**) The direction of stability loss only involves the *x*-axis; the transition starts along the *x*-axis but later involves the *y*-axis. (**d**) The direction of stability loss involves both the *x*- and the *y*-axis; the transition starts involving both the *x*- and the *y*-axis, but the *x*-value does not change much after the transition. (**e**) The direction of stability loss involves both the *x*- and the *y*-axes; the new attractor is not a point attractor but a limit circle, and both the *x* and the *y* values overlap with the previous point attractor

Unlike other verbal theories in psychopathology [31], the theory of EWSs has a formal background rooted in mathematical derivations, enabling the analysis of its key assumptions. The formal background of EWSs is based on bifurcation theory, which explains how a gradual change in a system parameter may lead to a qualitative change in the functioning of a system [6, 32–35]. We first use the case of a cusp bifurcation as a conceptual explanation, which is a rather simple scenario from bifurcation theory. Figure 1a (first row) shows a ball on a landscape with two local basins in different configurations (columns 1–7). The position of the ball represents the *state* of the system, the basins represent the *phases* of the system^3^, and the color represents the altitude of the landscape, showing the *stability* of the system^4^. In clinical cases, for example, the *x*-axis may represent mood, and the *y*-axis may represent sleep quality. The right basin could correspond to the healthy phase (i.e., high mood and high sleep quality), and the left basin to the depressive phase of a client (i.e., low mood and low sleep quality). When the basin is deeper (represented by darker blue colors), the phase is more stable; when the basin is shallower (represented by lighter blue and green colors), the landscape is shallower, and the phase is more unstable.

The shape of the stability landscape of the system is often determined by one or more *control parameters*. By adjusting the control parameter (which can be, e.g., alleviating financial stressors, improving social contacts, or making progress in psychotherapy), the landscape changes smoothly from the first column to the last column in Fig. 1a, resulting in a destabilization of the left, depressive basin and a stabilization of the right, healthy basin. At a certain point (termed the bifurcation point), the left basin no longer exists, causing the ball to abruptly move to the right basin, this occurs in the second to last column in Fig. 1a. This represents a qualitative transition in the state of the system, for example, a sudden gain in treatment. Before the transition, although the state of the system (i.e., the position of the ball) does not change much, the phase’s stability (i.e., the depth of the basin) decreases. As a result, when the system is under small perturbations, it is easier for the system to move to another position (i.e., the ball moves further away from the equilibrium point), and it is harder for the system to recover (i.e., the ball returns more slowly to the equilibrium point). For example, after experiencing positive events, the client becomes more joyful than before and does not return to the depressed state as quickly, which represents the destabilization of the depressed phase.

For real-life systems, it is often hard to tell how stable a phase exactly is (i.e., the altitude of the ball’s position is not easy to measure). However, if we observe that the state of the system (i.e., the position of the ball) exhibits increasing variance and increasing autocorrelation, we can infer that the stability of the phase is decreasing, and a critical transition may happen in the near future. Take a client with depression for example, it is difficult to exactly determine the client’s stability of the current depressive phase, yet we can assess how the mood level and sleep quality of this person change over time and infer the stability of the person’s depressive phase based on such statistical information.

EWSs do not only exist for the simple cusp bifurcation. Many real-life systems are similar to the cusp bifurcation in the sense that one basin of the system disappears at the tipping point [7], which means that there is at least one direction in which the system can tip over to a new phase.^5^ Mathematically, approaching the bifurcation point is asymptotic behavior, that is the autocorrelation of the system state will approach 1, and the variance of the system state will approach infinity (assuming small random perturbations). In real-life observations, it is of course not possible to observe this asymptotic behavior (i.e., we cannot use the local stability characteristics of the system to infer the dynamic properties when the system is *infinitely* close to the bifurcation point, and we cannot observe a system that is *infinitely* close to the bifurcation point while keeping the system from transitioning to another phase). Hence practically, the aforementioned conclusion implies that when the system is close *enough* to the bifurcation point, the primary factor that drives the changes in variance and autocorrelation is the destabilization of the current phase (i.e., the flattening of that valley in the potential landscape). The destabilization thus causes the increase in variation and autocorrelation, overshadowing the influence of other factors. This means that if we observe an *unusual* increase in variation and autocorrelation in a client’s symptom severity ratings (for instance calculated in a moving window), it is more likely that this increase is driven by the destabilization of the client’s landscape and that a sudden change is about to happen.

Previous researchers have provided mathematical proof for the presence of EWSs in one-dimensional systems [6] and multidimensional systems that can be sufficiently described by the tendency of descending along a landscape without involving curling forces [28]. Yet, real-life psychological systems are likely to be general multidimensional systems, for which the simplified assumptions above may not hold. In Supplementary Materials A, we conduct a mathematical derivation for multivariate systems in general, which is more realistic for real-life systems in the psychopathology domain and enables us to draw implications for multivariate research. As the details of mathematical derivation will not be informative for all empirical EWS researchers, we try to explain the gist of the theory in verbal form in the main text, with several specific scenarios shown with ball-and-landscape illustrations (the rows in Fig. 1). Those illustrations are all variants of the cusp model we depicted in the first row, Fig. 1a, with details explained in Supplementary Materials B. Using verbal descriptions and specific examples instead of mathematical derivations for general cases inevitably trades rigor for readability. Therefore, if a step is logically not strict enough in the verbal explanation or the generalizability of the specific scenarios is questioned, we refer the interested reader to the mathematical proof in Supplementary Materials A.

We start by introducing the assumed theoretical object of study as a multivariate stochastic dynamic system. This implies that there are multiple (cognitive, emotional, biological, social, etc.) variables that describe the mental state of an individual, and those variables have deterministic influences on each other, yet al.l those variables are also perturbed by random noise (e.g. everyday events). We also assume that for an individual diagnosed with a mental disorder, the dynamic interactions between the variables create a stable, system-wide attractor state that can be labeled as pathological (e.g., a depressive state) and that the system’s behavior remains in this stable state, even after some perturbations [12]. Consequently, it is difficult for the individual’s mental system to move far away from this pathological state. To simplify the subsequent discussion, we use the phrase the *strength of attraction* to refer to the strength of the pull exerted on the mental system of the individual to remain in the stable pathological state and the difficulty with which the system moves away from this stable state under perturbations.

If the strength of attraction remains high, the system is unlikely to escape from the pathological state. This must hold for all directions, which means that a small perturbation in *any* variable or any combination of variables cannot drive the system far away from this stable state. However, when the strength of attraction gradually decreases and approaches zero, a small perturbation can take the system away from this pathological state and make it transition to another state, which might be a healthier one. Just prior to the transition, the system still has the tendency to go back to the old stable state, but this tendency is weaker, so the speed at which the system returns to the old stable state is slower, which leads to an increase in the variance and autocorrelation of the system, known as the EWSs.

Here we emphasize the first important assumption of EWSs in multivariate systems: *the system should experience bifurcation-induced tipping*,* in which the system is attracted by a point attractor that loses its stability after the transition*. This means that before the transition, the system has the tendency to move to the single most stable *point*, but this tendency becomes weaker and weaker before the transition. Only then do variance and autocorrelation show the instability of the system. In a clinical scenario, a point attractor may correspond to a certain stable level of depressive symptomatology of a client. Sometimes the client may feel a little better or a little worse, due to all kinds of everyday events, yet the client always quickly returns to the same baseline level of depressive symptoms. Over the course of treatment, the depressed phase gets destabilized [12], which makes the client’s depressive symptoms fluctuate more. Also, little moments of feeling better may last longer and longer (i.e., the return time to the depressed state increases). Such increased fluctuations and return times are then EWS indicating that a sudden change will happen soon.

**Fig. 2:**
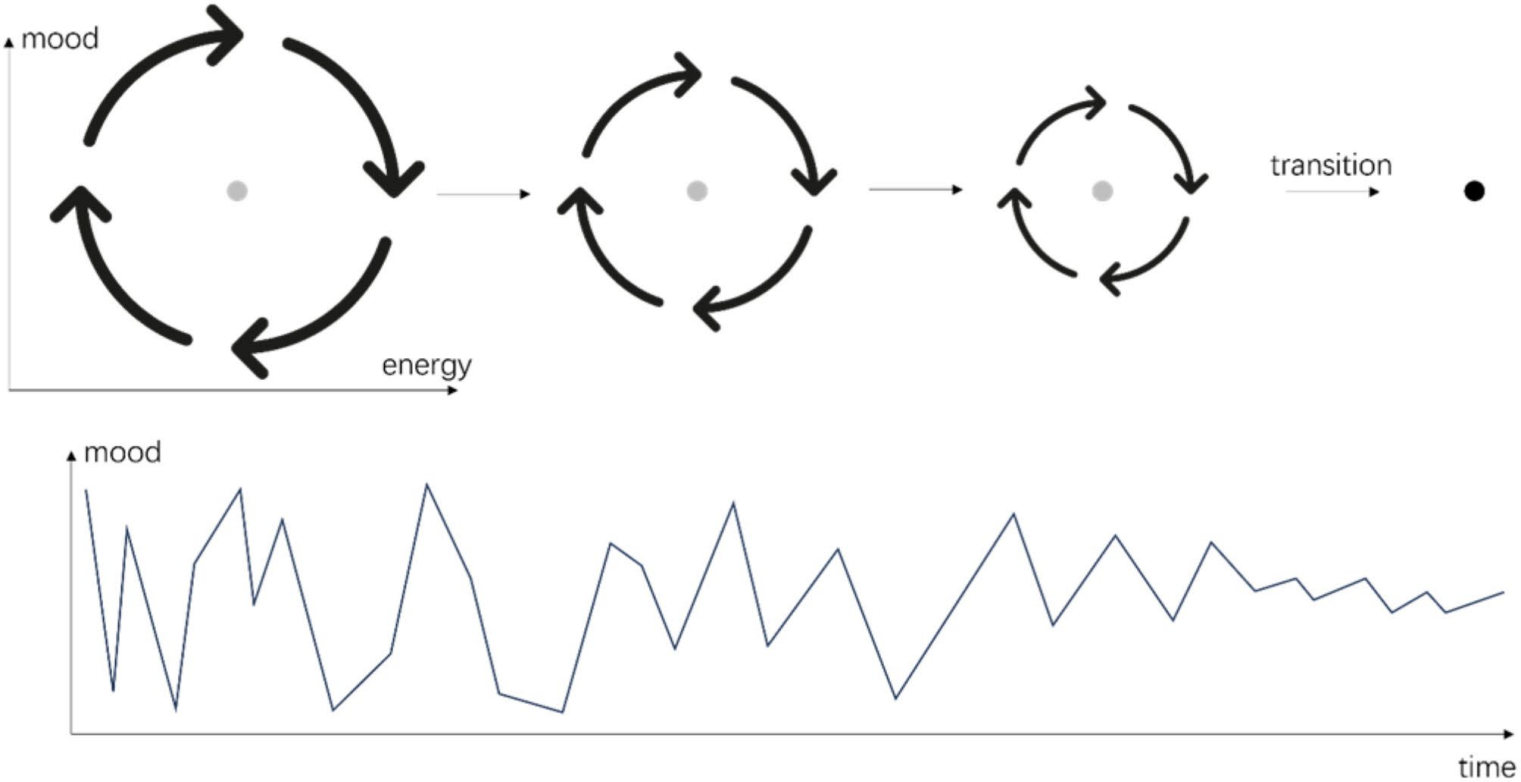
An example scenario without EWSs. For clients with rapid-switching forms of bipolar disorder or borderline personality disorder, the system transitions from a cyclic attractor to a point attractor. Thus, the variation and autocorrelation in mood measures represent the strength of fluctuation of the cyclic attractor instead of the stability of a point attractor

In all the ball-and-landscape scenarios in the rows of Fig. 1, the system starts from a point attractor (such as a stable level of depression). Note that in Fig. 1e, the system ends in a cyclic attractor, which is not a problem because what matters is that the system *starts* in a point attractor. After the transition, the system may end in a different type of attractor. This requirement is not to be taken lightly: if the system does not start in a point attractor, the variance and autocorrelation *will not* be indicators of the stability of the system. In Fig. 2 we depict a particular situation in which the system starts from a cyclic attractor. In this situation, the system tends to cycle between various states and the variance or autocorrelation of the variables represents the strength of the fluctuation instead of the instability of the fluctuating state. This type of situation may happen when cyclical fluctuations are an intrinsic property of the disorder, such as for rapid switching forms of bipolar disorder or borderline personality disorder [36]. In those clients, according to the model by Kraepelin [36], mood states are unstable and change rapidly, as well as energy levels (see Fig. 2, in which the horizontal and vertical dimensions represent the mood and energy of a client). In the beginning, a client may be in a high energy and manic state, then the energy of the person runs out, leading the client to a low energy and manic state. After that, the client goes to a low energy and depressed state, which leads to the cumulation of energy, and the client goes back to the high energy and manic state. The recovery of those clients manifests as the stabilization of the mood state and energy level, which means their mood and energy states become less extreme (i.e., the radius in the cyclic attractor of Fig. 2 becomes smaller). Thus, one would expect to find decreases in variance and autocorrelation of mood and energy measures in the case of successful treatment. Hence, the decrease of variance and autocorrelation does not mean that the client’s phase of bipolar disorder is becoming more stable because the attractor state of a (rapid switching) bipolar disorder, in terms of mood and energy variables, is not a point attractor. The system does not have the tendency to return to a single stable point but quickly alternates between different states. Therefore, it is not suitable to calculate EWSs for those clients based on mood and energy measures to use them for the prediction of sudden changes in their psychopathology, at least not from the methodological framework presented in the current paper.

The second assumption is that *the increase in variance and autocorrelation should appear closely before the transition*. This is already implied in the general concept of EWSs as the heralds of imminent change. The mathematical derivation that the variance will approach infinity and autocorrelation will approach 1 holds *only as* the system approaches the bifurcation point. As explained above, for real-life observation, this means EWSs overshadow other factors when the system is sufficiently close to the tipping point. If we calculate the variance or autocorrelation far before the transition, they are not able to pick up on rising instability among all other causes of variance and autocorrelation. If we calculate the variance or autocorrelation of the system during the transition, or after it has already transitioned to the new basin, the variance and autocorrelation of the variables are not, or not exclusively, related to the stability of the *previous* phase and may actually be relatively high as a consequence of the shift itself, instead of the rising instability. Taking the example of sudden gains in depression again, if we want to infer the stability of the depressive phase, we should not include observations of the healthy phase in calculations because anything calculated therefrom would become a representation of both phases instead of only the destabilization of the depressive phase. Therefore, it is only meaningful to use EWSs to predict a transition that is *about* to happen.

Note that we frame this assumption mainly for empirical research testing whether and when EWSs exist for psychological sudden changes. Practically, it would not make sense to calculate EWS after knowing that the transition has happened. But using EWS as a predictor in clinical practice is only possible after the phenomenon of EWS is sufficiently understood for clinical changes (i.e., after knowing when we can use what EWS to predict which kind of sudden changes for whom), which, according to our evaluation, is not fully established at the moment. It could be possible that, with sufficient empirical evidence, in the future, we know that a certain amplitude of EWSs is very likely to predict a clinical sudden change, which can then be used for practical purposes. Yet, this kind of empirical evidence needs to first be cumulated through studies with sound methodology, which is the core argument of the current article.

Besides the two assumptions mentioned above, there are some additional considerations for multivariate systems. For those systems, a key point is that a state is only stable if it is stable in *all* directions, but it is unstable if it is unstable in *any* direction. We again use the ball-and-landscape metaphor to describe this idea. Note that we can only show two variables, yet for real-life systems, there might be a much larger number of variables creating a multidimensional landscape. For a certain real-life transition, there are some directions^6^ in which the system loses its stability, and those directions may involve one or more variables. In Fig. 1a, this direction is to the upper right corner and involves both *x* and *y*, whereas, in Fig. 1b, this direction is parallel to the *x*-axis and only involves the variable *x*. In clinical cases, those directions may correspond to the symptoms that first start to appear or alleviate (we will provide more detailed examples later). The important point here is that even if it involves only one variable it still induces a leak in the basin, making the system transition out. Thus, our third assumption is that the direction in which the basin becomes flat is also the direction in which the system leaves the basin. Therefore, the direction of EWSs is also the direction of the start of the sudden change. In other words, *the EWSs and the start of the transition involve the same set of variables*^7^. Empirically, this simply implies that if a transition is evidenced in a (set of) variables, then the EWSs should be studied in the same variables, not in other simultaneously observed variables that do not show a transition. Note that the direction in which a system *starts a transition* does not necessarily map onto the straight line from the previous attractor to the new attractor. We illustrate this with Fig. 1c, in which the transition is along the *x*-axis at the beginning, but later also involves another variable *y*. Nevertheless, if one variable is involved in the destabilization of the system, then it must be involved in the transition process as well.^8^

Take two clients with depression for example. Assume the first one only has depressed mood as the major symptom (represented by the *x*-axis in Fig. 1a), and the second one has both depressed mood and sleeping problems (represented by the *x*- and *y*-axes in Fig. 1b or Fig. 1c). During treatment, both clients had a sudden gain and recovered. If we would monitor mood and sleeping problems across treatment, we may see a sudden change in mood for the first person, but sudden changes in both sleep quality and mood for the second person. In this case, we would expect the first person to have EWSs in mood before the transition, but not in sleep quality because the first person did not have sleep problems to begin with. For the second person, EWSs may occur in mood or sleep quality or both, because the person’s depressive phase may first destabilize along the sleep quality axis or the mood axis. If the person’s depressive phase first destabilizes along the sleep quality axis, (i.e., the person first improves in sleep which then also positively affects the mood), EWSs should be found in sleep quality but not mood, because changes in mood follow the initial destabilization in sleep. If the person’s depressive phase destabilizes along both axes simultaneously, EWSs should occur in both variables. If it is unknown on which axis the depressive phase first destabilizes, monitoring both variables for EWSs is best. There is no need to monitor other variables that are not a part of this person’s change process because these other variables (e.g. appetite) are not involved in the transition.

In sum, our mathematical derivation illustrates three important general assumptions for EWS research: (1) the system starts from a point attractor, and this attractor becomes unstable after the transition; (2) EWSs appear right before the transition, not after the transition or far before; (3) EWSs occur only in the variables in which the (start of the) transition occurs. These three assumptions are vital prerequisites for investigating whether EWSs exist or not as precursors to sudden changes in mental disorders. In the following sections, we further discuss the assumptions in relation to empirical studies and provide recommendations for future research.

### The first assumption: the system departs from a point attractor

As shown in our derivation, an important assumption of the underlying change mechanism is that the system before the transition is in a point attractor, but after the transition, that point attractor diminishes. Previous studies used different methods for detecting whether a sudden transition has happened, yet they do not always establish whether the transition departs from a point attractor (Table 1). Some studies used the difference in symptom severity scores before and after an assessment period (two measurement occasions) to indicate whether a transition had taken place. Using two measurement points does not provide enough information to investigate which type of attractor (point, cycle, or some other type) the system was previously in. Some other studies used several repeated assessments and examined if the change between successive assessment points exceeded a certain clinical threshold. The problem here is that if an assessment touches the threshold and then comes back to its previous value, it is still counted as a transition [24], even though the system did not enduringly leave the original phase. Helmich et al. [26] used a similar method, but with an additional requirement that the mean level difference before and after the identified transition had to be large enough, making the results more robust. Other researchers [19, 21] used change point analysis, which is a group of statistical methods seeking to find transition points in the time series. In general, those methods try to split the whole time series into parts to make the data points in each part relatively stable around their own means, but the mean value may differ significantly across different parts [22, 37]. The advantage of change point analysis is that it takes the whole time series into account, therefore includes more information, and it performs better in ruling out false positives because it only identifies a transition if the new phase is different enough from the previous one and also relatively stable. Change point analysis is powerful in detecting a point-to-point transition because it tries to make the data points stay close to the mean level, corresponding to the point attractor, before and after the transition. However, it may not work well for more complex transition types, for example, the point-to-circle transition shown in Fig. 1e. Traditional change point analysis used in most previous studies also only works for single-variate data, although multivariate extensions are available [38].

**Table 1:** An overview of the transition detection methods in previous studies

Transition detection method	Examples of empirical studies	Method details
Difference in two assessments (continuous; no criteria)	[30]	Difference of the HDRS-17 and SCL-90-R scores in the baseline and follow-up assessments
	[57]	Difference of the IDS-C score in the baseline and follow-up assessments
	[27]	Difference of the SCL-90-R score in the baseline and follow-up assessments
	[25]	Difference of the QIDS-SR score in the baseline and follow-up assessments
Difference in subsequent assessments above a threshold	[24]	An increase of ≥ 6 points in ASRM or QIDS-SR, without such increases in the previous two weeks. (The scores may decrease immediately after the transition.)
	[26]	A change that is more than a threshold determined by DaRCI (58) in the SCL-90-R depression subscale, with a stability check that there is a large change in the mean level before and after the transition.
Change point detection	[21]	Change point analysis (38) of the SCL-90-R depression subscale
	[19]	Change point analysis (59) of the item of problem intensity in TPQ

*Note.* Abbreviations: HDRS-17, Hamilton Depression Rating Scale; SCL-90-R, Symptom Checklist 90 Revised; IDS-C, Inventory of Depressive Symptomatology – Clinician Rating; QIDS-SR: Quick Inventory of Depression Symptomatology-Self Report; ASRM, Altman Self Rating Mania Scale; TPQ, Therapy Process Questionnaire

In summary, if it can be assumed that both attractors before and after the transition are point attractors, we suggest future researchers use change point analysis as a main approach for identifying critical transitions; if a threshold-based method is used, it is important to check if the system is stably residing in the new phase to rule out the influence of a single outlier. We also suggest a more general, descriptive method, namely the distance plot, to accompany the methods described above as a way to visually check whether there is a transition from a point attractor. A similar method based on recurrence plots has been proposed by [39], yet our method is more specific to check this specific assumption of EWSs. The distance plot is a two-dimensional plot that shows the Euclidian distance between system states at different points in time [40, 41].

$$\:{D}_{{t}_{i},{t}_{j}}=\left|\right|{\varvec{x}}_{i}-{\varvec{x}}_{j}\left|\right|,$$

in which the Euclidian distance for two vectors means the square root of the sum of squares of the differences in variable values. For example, if there are four variables assessed over time, and at time 1 the variables take the values of $\left(1,\:2,\:3,\:4\right)$, and at time 2 the variables take the values of $\left(2,\:3,\:2,\:3\right)$, then the Euclidian distance of the two time points is calculated by:

$$\begin{array}{l}\:{D}_{\text{1,2}}=\left|\left|\left(1,\:2,\:3,\:4\right)-\left(2,\:3,\:2,\:3\right)\right|\right|\\=\sqrt{{\left(1-2\right)}^{2}+{\left(2-3\right)}^{2}+{\left(3-2\right)}^{2}+{\left(4-3\right)}^{2}}=2.\end{array}$$

**Fig. 3:**
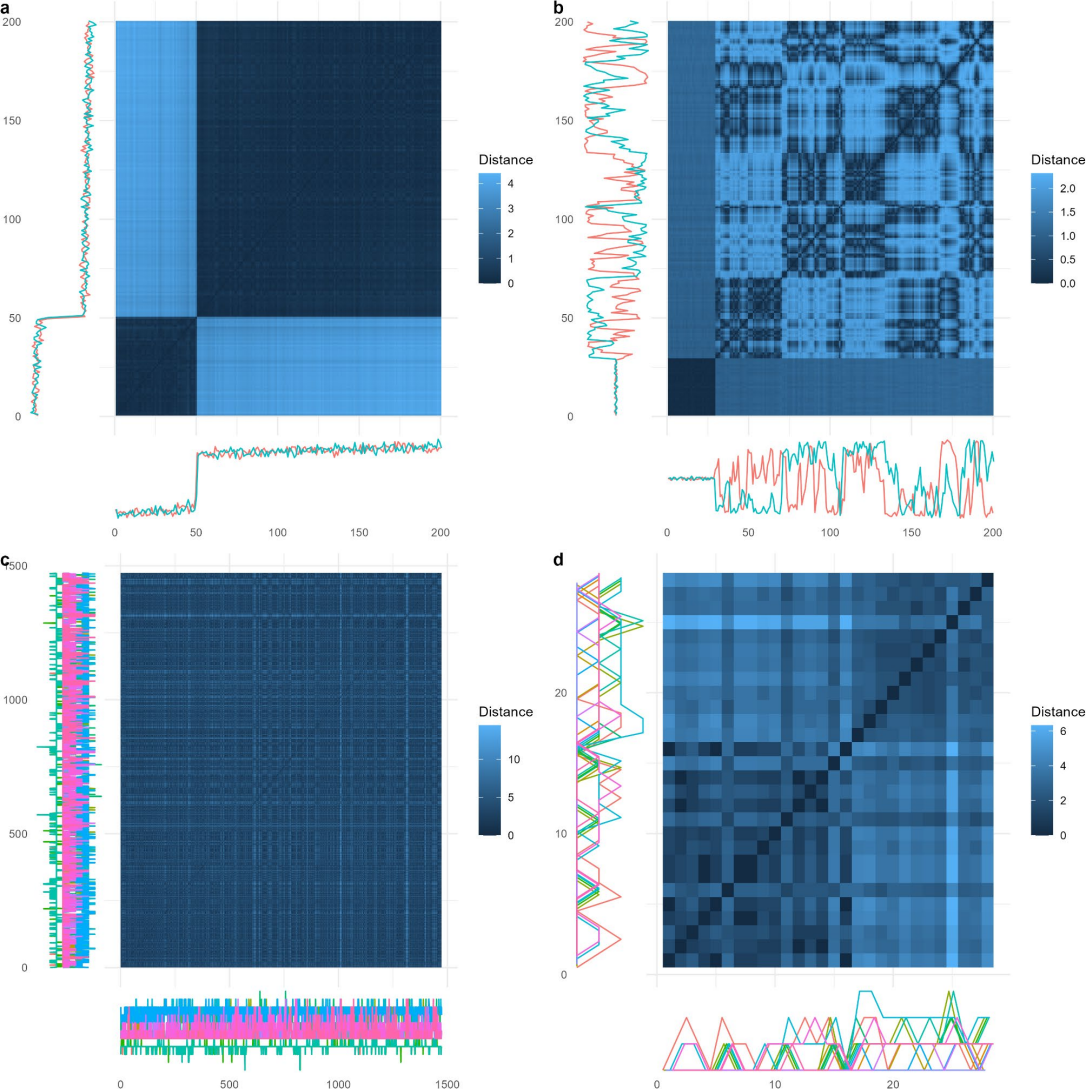
Distance plots for (**a**) simulated system based on the example in Fig. 1a, (**b**) simulated system based on the example in Fig. 1e, (**c**) ESM affect items, and (**d**) item scores of the depression subscale of SCL-90-R from [42]

If the system is attracted by a point attractor, under a small perturbation, the system will fluctuate closely around this point. Therefore, the distance between each pair of observations should be relatively small. However, after the transition, the system no longer stably moves around this point attractor. Its state is either far from the point attractor or only transiently crosses the point attractor. In a distance plot, both the horizontal axis and the vertical axis represent time, and each pairwise Euclidian distance of points is shown as the color of a pixel. The pixel color in column 1 and row 2 of the matrix, for example, represents the magnitude of the Euclidian distance between the state of the system observed at time 1 compared to the state at time 2. Since the distance between the two points is the same regardless of the temporal order (i.e. time 2 compared to time 1), a distance plot is always symmetric around the diagonal line. If there is a dark region on the plot, it would indicate the time points along it have similar values. In contrast, if there is a light region, it would indicate the time points along it have very different values. Therefore, if the system leaves a point attractor, the distance plot will show a square of dark region before the transition, and a light rectangle of rather far distance next to the square. In Fig. 3a-b, we show the distance plots for the bivariate simulated data from examples of point-to-point transition and point-to-circle transition in Fig. 1a and e (with the raw time series shown along the axes; a description of the simulation procedure can be found in Supplementary Materials B). From the results, it is clear that the method can detect the system leaving a point attractor, no matter the shape of the new attractor. In Fig. 3b, the new phase does not show a square, but different blocks, which indicates a non-point attractor. This is not a problem because what is important in the theoretical assumption is about the attractor *before* the transition, not *after* the transition.

We also apply this method to an empirical dataset described and made open access by [42]. This dataset is from a client with major depressive disorder (MDD) who completed daily ESM measures up to 10 times per day for 239 consecutive days. Besides that, the participant also completed the depression subscale of the Symptom Checklist-Revised (SCL-90-R). For illustration, we use the time series of 13 ESM affect items^9^ used in [20] and the item scores from the depression subscale of SCL-90-R (28 time points; *M* = 1.57, SD = 0.44). The distance plots for both datasets are shown in Fig. 3c-d. From the results, it becomes clear that the symptom severity of the system assessed by the depression subscale of SCL-90-R may have experienced a transition out of a point attractor, but the ESM affect items did not. Therefore, the critical transition is more evident in the symptom severity scale instead of the ESM affect measures. We will further elaborate on this point when discussing the third assumption. Note that, although differences in time intervals between observations are an important issue to solve for dynamic statistical models, it is of minor relevance for the inferences we can draw from the distance plot as long as the total observation time is sufficiently long to capture a potential transition. This is mainly because the distance plot is used to detect the critical change that is evident from the whole time series instead of dynamic relationships between consecutive time points.

### The second assumption: EWSs appear before the critical transition

**Fig. 4:**
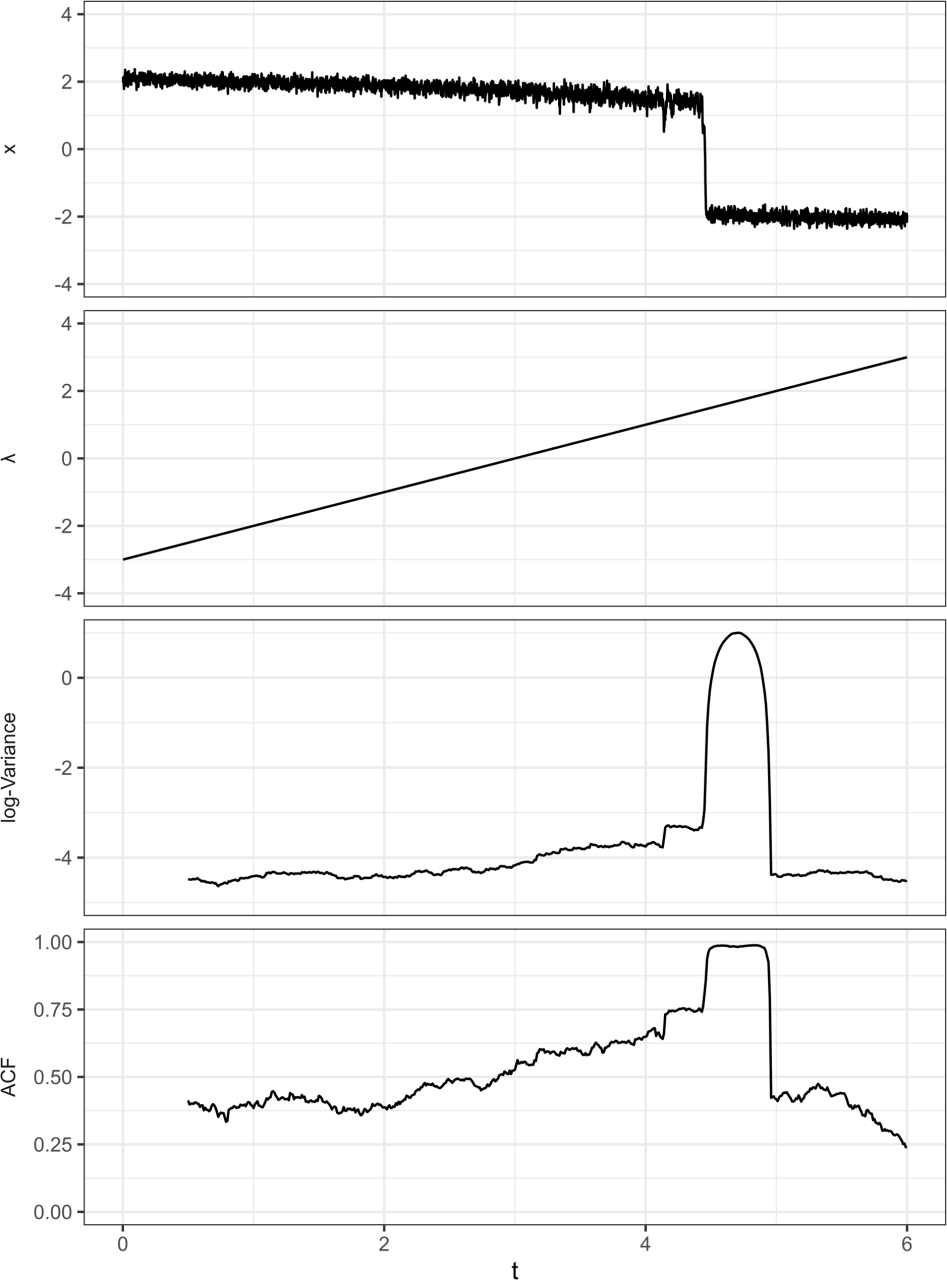
A simulation example of a transition with EWSs. The variance and ACF increase before the transition, which are the true EWSs. However, the transition itself creates an even higher peak in variance and ACF, which are not EWSs. Those increases after the transition may be mistakenly taken as evidence of EWSs if the time window of calculation is not strictly before the transition. See Supplementary Materials B for details of the simulation setup

As shown previously, EWSs should appear before the sudden transition and predict a forthcoming transition. This is also a conceptual and practical requirement if we want to identify *early* warning signals to predict future sudden transitions. For empirical studies *validating* the existence of EWSs, it is also crucial to make sure EWSs are calculated before the transition because the transition itself can also lead to an increase in variance and autocorrelation which is not an *early* warning signal (see Fig. 4 for an example and see Supplementary Materials B for a simulation study). The influence of the transition itself on EWSs persists even after detrending the time series, a standard procedure in EWS calculation [20] (the time series in Fig. 4 was also detrended before EWS calculation). Although detrending can remove the effect of gradual trends, it is less effective for a sudden change. Looking into previous literature, we found that although some studies explicitly make sure EWSs were calculated before the transitions, it is not always the case for other studies (Table 2).

**Table 2:** An overview of assessment periods of early warning signals in previous studies

EWS calculation period	Examples of empirical studies	Details
Around the transition	[25, 30, 57]	During the whole assessment; between-participant comparison
Roughly before the transition	[24]	Before the time that the transition was detected, including the week before the sudden increase
Strictly before the transition	[20, 21, 26, 27]	Before the time that the transition was detected, excluding the week before the sudden increase
	[19]	Before the time the transition was detected (the variables used for transition detection were assessed daily)

In order to examine possible EWSs strictly before the transition, the first prerequisite is to rigorously pinpoint the moment of the transition in empirical studies. Some studies calculate EWSs for the whole assessment period, without identifying the exact moment of the transition. For example, the study by [30] used the difference in depression score before and after treatment (for depressed clients) or observation period (for the general population) to represent whether a transition had taken place or not. Results of this study showed that participants with a greater change in their depression scores also had a larger correlation between emotion scores. However, because depression scores were not measured repeatedly throughout the study period, it was not possible to distinguish between a critical transition and a gradual change [43], and even if we assume that a sudden change took place, it is impossible to identify the exact time point of that transition. Consequently, if a transition had taken place, the EWS calculation period could have included the transition.

For studies in which the timing of the transition is identified, it is still possible that the EWS calculation period involves the transition. For example, in the study by [24], the variables used for detecting transitions were measured weekly, but the data used for calculating EWSs was measured daily up until the transition point. Therefore, the time window for calculating EWSs may also include the transition point because the transition may have happened anytime during that week. In that case, the transition itself would lead to an increase in variance and autocorrelation. The EWSs detected may then not be true EWSs, but rather, a statistical byproduct of the transition. For many other studies using the same assessment frequency [24, 26, 27], the week before the detected transition was excluded from EWS calculations. In those cases, you move further away from the transition point and lose information by excluding the prior week but at least it is certain that the EWSs were calculated strictly before the transition. For the studies using the same assessment frequency for all variables [19], there is no such pitfall, so it is relatively straightforward to make sure EWSs are calculated before the transition.

For future research that aims to *validate* the existence of EWSs, our suggestion is that EWSs should always be calculated in a period preceding the transition. At the same time, we do not recommend discarding data before and close to the transition or assessing EWSs too early before the transition because EWSs are the most salient right before the transition. Therefore, we suggest the best approach to take is trying to detect the transition with high time precision, preferably as frequently as all other measures in the study. When that is not possible, researchers should try to make sure that the EWS assessment period does not include the transition, even if it is further apart from the transition.

We would also like to note that the implication of this assumption for EWS *validation* studies is naturally different for the research that aims to *apply* EWSs in clinical practice. The application of EWSs in clinical practice is ideally undertaken after the predictive power of EWSs is validated to a certain degree. To our knowledge, most of the studies in the past decade are validation-type studies, and we suggest given the current status of the field, further validation studies are still needed before real-life applications. Therefore, our investigations in this section are mainly intended for validation studies. In validation studies, often the whole observation period is recorded and analyzed afterward which means that the transition point is known and can be pinpointed in the time series. Once EWSs have been validated we can move to the application scenario in clinical practice. Here, the patient’s mental state is monitored in real time not knowing when a transition is going to happen. When EWSs are detected (e.g., alleviated variance and autocorrelation of the time series exceeds a threshold), the therapist can take certain measures to prevent an undesirable transition or to promote a desirable transition. This action should be taken, theoretically, before the transition, thus what is observed is that the patient is still in the previous phase but showing EWSs of an upcoming transition. In this case, EWSs are naturally calculated before a possible transition to meet this assumption.

### The third assumption: EWS variables align with system destabilization

As shown in the previous derivation, the variables that show EWSs are the same ones that point to the direction of destabilization. In other words, it is the same set of variables that show EWSs and that are involved in the start of the transition. This does not mean that the variables that show sudden transitions *always* have preceding EWSs (see Fig. 1c and [44]), because EWSs only show the direction in which the system loses its stability at the *beginning*. After leaving the previous stability basin, it is possible that the system changes its direction and involves more variables. Nevertheless, as there is not enough evidence, to our knowledge, to specify which variables are likely to only show transitions without EWSs, it is more reasonable to calculate EWSs and detect transitions for all variables of interest. In other words, there is no reason to *exclude* the variables that have sudden transitions from the calculation of EWSs, while there are good theoretical reasons to include them (see Supplementary Materials B for a simulation study showing the possible consequences of excluding variables with sudden transitions from EWS calculations).

In previous studies on clinical EWSs, however, we found that it is very common to exclude the variables for sudden change detection from the EWS calculation (Table 3). Actually, no studies that we found in the field of clinical psychology have any shared variables for detecting EWSs and sudden changes. Some of them used different scales for the two sets of variables. For example, in the study by [20], SCL-90-R was used for detecting sudden changes, whereas the ESM affect measures were used to detect EWSs. As shown previously, the ESM affect measures of this dataset did not show a clear transition during the assessment period, which means that the sudden change was not evident enough from the ESM measures of the client. The EWSs calculated for those variables are therefore possibly unrelated to the destabilization before the transition in symptom severity.^10^ Some studies used a single scale for both assessments but excluded the variables that were used for detecting sudden changes for calculating EWSs [19]. This kind of exclusion is unnecessary and makes it difficult to tell if the variables that undergo the transition also show EWSs. In Table 3, we show several examples in other fields (e.g., physics, ecology, and movement science). Actually, we did not find any studies in other fields, from which EWS studies in clinical study drew much inspiration, that separate the variables into two sets, one for transition detection and another for EWS calculation. Therefore, we suggest future researchers consistently use the same variable(s) to detect transitions and EWSs.^11^

**Table 3:** An overview of the variables used in EWS studies in clinical psychology and other fields of complex systems

Same variable(s)	Examples of empirical studies	Variable(s) of sudden change	EWS
No	[30]	HDRS-17 or SCL-90-R	Variance, ACF, cross-correlations of ESM measures of four emotions
	[20]	SCL-90-R	Variation, ACF, cross-correlations of ESM measures of affect
	[19]	The item of problem intensity in TPQ	The dynamic complexity of all other variables in the TPQ
	[25]	QIDS-SR	Variance, ACF, cross-correlations of 10 items from the PANAS
	[24]	ASRM and QIDS-SR	ACF of 17 items of momentary mood and symptoms
Yes (examples from other fields)	[60]	Light absorption	Variance of light absorption of different experiment trials
	[61]	Laser intensity	The time that laser intensity reaches the second phase
	[62]	Temperature and deuterium concentration	ACF of temperature and deuterium concentration
	[63]	EEG channel activity	Variance of EEG channel activity
	[64]	Phase modulus of finger movements	Variance of phase modulus of finger movements

*Note.* We did not find empirical studies in clinical psychology that use the same set of variables for sudden change detection and EWS calculation. Therefore, we included several studies in other fields, selected from [7], as examples. Abbreviations: HDRS-17, Hamilton Depression Rating Scale; SCL-90-R, Symptom Checklist 90 Revised; TPQ, Therapy Process Questionnaire; QIDS-SR, Quick Inventory of Depression Symptomatology-Self Report; ASRM: Altman Self-Rating Mania Scale; PANAS, Positive and Negative Affect Schedule

Determining which variables to measure and how to measure them is a non-trivial question, especially in the field of ESM [47]. Take the affect measures used in [20], items such as “I feel down” are in the literature used as a symptom, emotion, affective state, or mood measure. This blurry distinction in the field of ESM studies makes it difficult to compare studies and look for converging evidence. A general strategy for choosing the “right” variable cannot be given but depends on the research question and the phenomenon of interest. If one is interested in transitions in symptom severity, then symptom severity should be tracked over time to determine the transition and look for EWS. If the focus lies on transitions in mood, then mood is what needs to be measured. Once that more conceptual decision has been made the assessment procedure should be adjusted accordingly, that is the measurement frequency should match the phenomenon of interest and the phrasing of the question should be explicit about the type of process the item is tapping into. More concretely, if the researcher is interested in momentary affect transitions, the measurement frequency should be dense enough to be able to pick up on transitions at this rather fast time scale and the question asked should make clear that respondents are asked to reflect on their momentary affective state (e.g. “I feel down *now*” rather than “I feel down”). Another complicating factor in the field of psychology is that different people may interpret certain questions differently and may vary in their response tendencies (across people and within people over time). For this issue, an idiographic approach, where patients collaborate with their therapists to identify personally meaningful items in their lives, represents a promising strategy [46].

## Discussion

This article aims to examine the current research methodology in clinical EWSs from a theoretical perspective. The motivation for this examination comes from the variety of methods and mixed research findings in this field and also from the nature of theory-based prediction of EWS studies. In order to find out which study methods and designs are the most suitable for studying clinical EWSs, we first investigated the theory of EWS for multivariate systems using a mathematical derivation. From the theoretical investigation, we identified three key assumptions used during the derivation: (1) the system starts from a point attractor that disappears after the critical transition, (2) early warning signals appear strictly before the critical transition, and (3) the same set of variables are involved both in EWSs sudden changes. Based on those assumptions, we evaluated common practices in recent literature on EWSs in clinical psychology and found that those assumptions were not always met or examined in empirical studies. Finally, we provided suggestions for future empirical studies.

The strength of our approach lies in the close alignment of methods and theory. All the evaluations and suggestions proposed are grounded in the mathematical derivation of early warning signals in general multivariate dynamic systems, drawing from the foundation of bifurcation theory. This imparts a robust framework to guide future investigations. However, we acknowledge the essential need for a critical examination of our theory’s real-world applicability. In complex, real-life systems, various forms of changes may exist [48–50]. These changes may give rise to different statistical indicators and require different investigation methods, yet they have remained relatively understudied within the field of clinical psychology [9]. We advocate for a more dedicated exploration of the essence of these transitional phenomena in future research. Only by shedding light on less-explored facets can we ensure that our statistical advancements yield practical utility and relevance.

The three key assumptions presented here are certainly not sufficient to guarantee the detection of EWSs. We highlighted them in this article as we consider them the most salient ones in the current methodological context of the field and thus warrant specific attention. All three assumptions are important and do not have trade-off relationships, thus the best scenario would be to ensure that all three assumptions are met. Practically, however, we can see that researchers may not always be completely certain, especially for the first and the second assumptions which are usually bound to the accuracy of visual checks and statistical inferences. In contrast, the third assumption can be met with certainty by methodological choice. Therefore, we encourage researchers to always check whether the third assumption is met, while for the other two, transparent reporting about related procedures and communicating how likely it is that those assumptions are met might suffice in uncertain situations.

Besides the conditions discussed in this article, several recent studies have pointed out other important conditions for EWSs, such that the noise in the system should be Markovian white noise [51, 52], that the variables of interest should be sufficiently sampled with adequate accuracy [28, 29], that both variance and autocorrelation should increase instead one of them [53], and so on. While a detailed summary of all the important methodological considerations is out of the scope of the current article, we encourage methodological researchers to continue the investigation of the applicability of those conditions in the psychopathology field and search for ways to improve the detection of EWSs, and we encourage empirical researchers to actively follow the methodological advances in the field and apply available assumption checking methods in their data.

In this article, we examined whether the current research methodology in clinical EWSs is aligned with the theory, and we provided suggestions according to the theory. EWSs are essentially theory-based techniques, and we would like to emphasize the meaning and value of theory-based prediction. There is no guarantee that a theory-based approach can always predict sudden changes in clinical trajectories successfully or even have better predictive power than methods that are not well aligned with theory. It is possible that another prediction method, for example, a machine-learning-based approach [54] or outlier detection methods [55], yields a better prediction precision than EWSs. Still, the merit of theory-based predictions lies in several other aspects. First, we can gain a better understanding of the transition process itself and use the results to enhance theory formation. In EWS studies, for example, we start with the theoretical hypothesis that sudden gains and sudden losses in clinical psychology can be conceptualized as bifurcations. From there, we deduct the inference that EWSs should exist with certain assumptions. Therefore, rigorous examination of EWSs within a strong theoretical framework can provide evidence consistent or inconsistent with the hypothesis that clinical transitions are driven by bifurcation tipping. However, because EWSs may also arise in other types of changes, their presence or absence should be interpreted in combination with additional theoretical and empirical considerations (see [9] for detailed discussion), rather than being taken as definitive evidence. If bifurcation as the change mechanism is supported, some control parameters may exist for such bifurcation-induced changes. Those control parameters have important clinical implications because they are the key reason why a client becomes stuck in a maladaptive phase and, therefore, the key intervention targets. At the same time, they may not be as salient as the symptoms themselves and thus can be overlooked if the treatment only focuses on symptoms. Further research may be conducted to determine the underlying control parameters that determine the stability landscape of the system, which might be related to stressors or vulnerability in general or more specific factors for an individual or their social, economic, or cultural environment [14], and find ways to intervene on the control parameters, thereby benefiting intervention science. Only knowing that a statistical indicator can predict a forthcoming transition accurately without a theoretical reason, on the other hand, does not increase understanding of the clinical change processes. Second, with theory-based predictions, we can understand in which case the prediction method is generalizable, and how reliable it is. A theory states under which conditions a conclusion can be made, whereas statistical predictors do not specify those. In fact, without a theory, it is questionable if any conclusions based on the sample can be generalized to a new situation [56].

In summary, the investigation of EWSs in clinical settings represents a promising research direction that has the potential to significantly enhance our understanding of change mechanisms in mental health and psychopathology and ultimately inform intervention science. While empirical evidence is crucial, it is equally important to prioritize theoretical refinement and methodological evaluation within the field. Such efforts will not only drive theoretical advances but also lay the foundation for a more accurate and responsible use of EWSs in real-life practice, which is the ultimate goal that researchers in this field aspire to achieve in the future. By fostering a synergistic relationship between theory, methodology, and empirical work, we can strive for comprehensive progress and advance the field of clinical EWSs toward improved well-being for individuals.

## Conclusions

EWSs are theory-based prediction tools and require a good alignment of the methodology and the underlying theory. Based on our investigation of EWS theories and research practices, we found that the popular research designs in the field do not always align with the theory, leaving room for improvement. We suggest future research in clinical EWSs should be designed so that the transition is visually checked with distance plots, the EWS assessment period is strictly before the transition, and a consistent set of variables is used.

## Electronic supplementary material

Below is the link to the electronic supplementary material.

Supplementary Material 1

Supplementary Material 2

## Data Availability

The necessary code for replicating the findings presented in this article can be obtained from the following URL: https://osf.io/f659u/.

## Footnotes

1 Here we use the term “phase” to represent a collection of system states that are related to a system attractor and have similar qualities. Other literature may use different terms for a similar concept, whereas we aim to make clear the differences between a single state and a collection of states and stay consistent with our previous work [8, 9].

2 A related, well-studied concept is emotional inertia, which is also operationalized as high autocorrelations (specifically in affective states). Conceptually, emotional inertia refers to the persistence of feelings across time and contexts, or the inability of affective states to be adaptively regulated to baseline after perturbations, and a large body of empirical findings has suggested that high emotional inertia is related to psychological maladjustments and various mental disorders [16–18]. Yet recently, Koval and Kuppens [17] proposed to understand emotional inertia as EWSs for sudden loss based on both conceptual and empirical evidence. Both emotional inertia and EWSs can show the inability to return to the stable attractor (also known as the adaptive home base in some literature) after perturbations and can be assessed through autocorrelation in individual time series. Moreover, most consistent findings regarding emotional inertia were found by comparing healthy individuals with different vulnerability levels instead of comparing people with and without mental disorders, or people with different severity levels of mental disorders. Therefore, high emotional inertia is more likely to be a warning sign for healthy individuals instead of a general indicator of mental illness. Taken together, emotional inertia may be a special case of EWSs indicative of maladaptive transitions. In other words, emotional inertia can be used as an indicator of vulnerability in healthy individuals, but in general, emotional inertia may be better conceived of as a signal of change instead of a maladaptive characteristic. We would like to refer readers interested in the relationship between emotional inertia and EWSs to [17], for more comprehensive discussion. Although there is a close link between the two concepts, most studies under the term emotional inertia are based on interindividual comparisons instead of studying whether emotional inertia increases within an individual. Consequently, those studies aim to detect who is likely to have mental disorders instead of when a person is likely to have a sudden change. In the current article, we focus on the studies under the term EWSs, whose primary focus is to predict whether a sudden change is likely to happen in the near future within an individual.

3 To avoid confusion, the term “state” is used in this article for the specific condition defined by the values of the system variables, and “phase” is used for the higher-level patterns of the system, which consist of many states that are qualitatively similar [8].

4 Note that the definition of “stability” in this article may not be the same as some other works in this field (e.g., the time that a system spends to go back to its local minimum after a small perturbation [28]),. See [8] for the relationships between different stability measures based on a stability landscape.

5 In mathematical language, this means the dominant eigenvalue at that equilibrium becomes zero.

6 Mathematically, those directions are pointed by the leading eigenvectors.

7 Mathematically, this implies that only variables with nonzero loadings in the eigenvector will exhibit variance approaching infinity and autocorrelation approaching 1 as the system approaches the bifurcation point. Detailed illustrations can be found in Supplementary Materials A.

8 A special case is that the new state may have a similar value on an axis even if the system destabilized along this axis before the transition (e.g., the x-axis inFig.  1d). Therefore, if we only observe the system before and entirely after the transition, we may not be able to observe the change in this axis during the transition. This may happen in real life, for example, if the *x*-axis represents the external behavior of a child, and the *y*-axis represents the conflict resolution of parents. If the conflict resolution of parents is inferior, the child may not dare to exhibit externalizing behavior to prevent aggravating the conflicts; when the conflict resolution of parents improves, the child may start to exhibit externalizing behavior; when the conflict resolution of parents improves even more, the externalizing behavior of the child may reduce again to a level similar to the beginning, but now the whole family system is much healthier. In this case, the child’s externalizing behavior may show EWSs before the transition even if the externalizing behavior’s beginning and end levels are similar. Nevertheless, if we observe the whole trajectory, it would be obvious that the child’s externalizing behavior also plays a role and changes through the transition, so our conclusion still holds in this case.

9 Those items include irritated, content, lonely, anxious, enthusiastic, cheerful, guilty, indecisive, strong, restless, agitated, suspicious, and worries.

10 Wichers et al. [20] conducted two sets of assessments of the client, one is ESM measures of momentary affects, and the other is weekly, retrospective measures of symptom severity. Whereas the symptom severity time series showed a clear sudden change, there was no clear sudden change in the ESM measures. Wichers et al. [20] claimed that they found evidence of EWSs in ESM measures, but based on our argument, it is unclear whether it is related to the sudden change in symptom severity. As the number of assessments of symptom severity is too few in this dataset (15 data points before the transition) and typically considered unsuitable for EWS calculations, we could not examine whether EWSs were present in the weekly symptom measures.

11 There are several methods designed specifically for multivariate EWSs [45] and some of them (e.g., PCA-based approach [23, 46]), have been used for psychological data. Those methods also have the potential to be incorporated into the theoretical framework we proposed. Although providing derivations for those methods is out of the scope of the current article, we encourage future research to take this further step.

## Abbreviations

EWS: Early warning signal
ESM: Experience sampling method
MDD: Major depressive disorder
SCL-90-R: Symptom checklist-revised
