# Supplementary material for: Examining the research methods of early warning signals in clinical psychology through a theoretical lens
Source: BMC Psychiatry. 2025 Mar 19;25:261. doi: 10.1186/s12888-025-06688-5 (PMC11924765; doi:10.1186/s12888-025-06688-5)
Supplement: Supplementary file 1 — Supplementary Material 1 [file 12888_2025_6688_MOESM1_ESM.pdf]

Supplementary Materials A: Formal Derivation of the EWS Theory for Multivariate Systems

For the manuscript "Examining the Research Methods of Early Warning Signals in Clinical Psychology through a Theoretical Lens"

In this part, we aim to provide a formal derivation of the existence of early warning signals for general multivariate systems and systematically examine the assumptions involved in the derivation. The proof for one-dimensional systems and multidimensional systems with symmetric Jacobian has been given in previous work (Dablander et al., 2022; Scheffer et al., 2009).

Assume the dynamic system is governed by the following ordinary differential equation:

$$\frac{\mathrm{d}\,\boldsymbol{x}}{\mathrm{d}\,t} = \boldsymbol{F}(\boldsymbol{x}), \tag{1}$$

and assume there is an equilibrium point $\boldsymbol{x}_0$ that $\boldsymbol{F}(\boldsymbol{x}_0) = \boldsymbol{0}$. To simplify notations, we assume $\boldsymbol{x}_0 = \boldsymbol{0}$. When the system is close enough to the equilibrium point, with local linearization, the dynamics can be represented by a linear Jacobian matrix:

$$\frac{\mathrm{d}\,\boldsymbol{x}}{\mathrm{d}\,t} = \boldsymbol{J}\boldsymbol{x}, \tag{2}$$

where $\boldsymbol{J} = \begin{bmatrix} \frac{\partial F_1}{\partial x_1} & \cdots & \frac{\partial F_1}{\partial x_n} \\ \vdots & \ddots & \vdots \\ \frac{\partial F_n}{\partial x_1} & \cdots & \frac{\partial F_n}{\partial x_n} \end{bmatrix}$, n is the dimension of the system, F_i and x_i represents the i-th component of $\boldsymbol{F}$ and $\boldsymbol{x}$. Here, the local linearization means that we assume the force that pulls the system back to (or pushes the system away from) the local minimum is proportional to the distance it is from the local minimum. Holding the same direction, the further the system is from the local minimum, the stronger the force to pull it back (or push it away).

The forces that pull the system back (or push it away) depend on the direction in which the system is away from the local minimum. There are specific directions that characterize the effect of the force, represented by the *eigenvectors* of $\boldsymbol{J}$. Each eigenvector also has an eigenvalue, representing the strength of the force. The eigenvalue may be complex numbers, but whether the force tends to pull the system back or push the system away only depends on the real part of the eigenvalue. Because the equilibrium point is stable, the system should be pulled back under a small perturbation, no matter in which direction. This means all the eigenvalues of $\boldsymbol{J}$ should only have negative real parts. The eigenvalue with the largest real part (the eigenvalue with the smallest absolute value of the real part) is called the dominant eigenvalue (λ_d). So the equilibrium condition is equivalent to $\mathrm{Re}(\lambda_d) < 0$. We first show the derivations with the assumption that all eigenvalues and eigenvectors of $\boldsymbol{J}$ are real values. The situation with complex eigenvalues and eigenvectors has similar conclusions and will be discussed later. When the system is close to the catastrophic bifurcation point λ_d approaches zero, making the system gradually unstable in this direction. This instability manifests when we add noise in the system, as represented by the following multivariate Ornstein-Uhlenbeck process,

$$\mathrm{d}\,\boldsymbol{X}(t) = \boldsymbol{J}\boldsymbol{X}(t)\,\mathrm{d}\,t + \boldsymbol{\sigma}\,\mathrm{d}\,\boldsymbol{W}(t). \tag{3}$$

To simplify the derivation, we first do a transformation of the coordinates with eigenvalue decomposition.

$$\mathrm{d}\,\boldsymbol{X}'(t) = \boldsymbol{\Lambda}\boldsymbol{X}'(t)\,\mathrm{d}\,t + \boldsymbol{\sigma}'\,\mathrm{d}\,\boldsymbol{W}(t), \tag{4}$$

where $\boldsymbol{X}'(t) = \boldsymbol{Q}^{-1}X(t)$, $\boldsymbol{\Lambda} = \boldsymbol{Q}^{-1}\boldsymbol{J}\boldsymbol{Q}$, $\boldsymbol{\sigma}' = \boldsymbol{Q}^{-1}\boldsymbol{\sigma}(t)$. Here $\boldsymbol{Q}$ is a square matrix whose columns are the eigenvectors of $\boldsymbol{J}$, and $\boldsymbol{\Lambda}$ is a diagonal matrix whose diagonal elements are the eigenvalues of $\boldsymbol{J}$. The order of the eigenvalues in $\boldsymbol{\Lambda}$ is arbitrary. Here, we assume that the first eigenvalue is the dominant eigenvalue, and it is the only eigenvalue that will approach zero from the negative side ($\lambda_1 \to 0^-$). Now we investigate the variance and covariance of the process. It can be proved that Ornstein-Uhlenbeck processes always have a multivariate normal distribution, and the variance of the Ornstein-Uhlenbeck process above for a sufficiently long time can be calculated by the following formulas (Meucci, 2009; Vatiwutipong & Phewchean, 2019),

$$\mathrm{vec}\left(\boldsymbol{\Sigma}'_\infty\right) = \left[-(\boldsymbol{\Lambda} \oplus \boldsymbol{\Lambda})\right]^{-1} \mathrm{vec}(\boldsymbol{\Sigma}'), \tag{5}$$

where $\mathbf{\Sigma}' = \boldsymbol{\sigma}'\boldsymbol{\sigma}'^T = \boldsymbol{Q}^{-1}\mathbf{\Sigma}(\boldsymbol{Q}^{-1})^T$, vec is the vectorization parameter that transforms a matrix to a column vector by stacking the columns of the matrix together and $\oplus$ is the Kronecker sum defined by

$$\begin{aligned}
&\begin{bmatrix} a_{11} & \cdots & a_{1n} \\ \vdots & \ddots & \vdots \\ a_{n1} & \cdots & a_{nn} \end{bmatrix} \oplus \begin{bmatrix} b_{11} & \cdots & b_{1n} \\ \vdots & \ddots & \vdots \\ b_{n1} & \cdots & b_{nn} \end{bmatrix} \\
&= \begin{bmatrix}
a_{11}+b_{11} & \cdots & b_{1n} & \cdots & \cdots & a_{1n} & \cdots & 0 \\
\vdots & \ddots & \vdots & \ddots & \ddots & \vdots & \ddots & \vdots \\
b_{n1} & \cdots & a_{11}+b_{nn} & \ddots & \ddots & 0 & \cdots & a_{1n} \\
\vdots & \ddots & \ddots & \ddots & \ddots & \ddots & \ddots & \vdots \\
\vdots & \ddots & \ddots & \ddots & \ddots & \ddots & \ddots & \vdots \\
a_{n1} & \cdots & 0 & \ddots & \ddots & a_{nn}+b_{11} & \cdots & b_{1n} \\
\vdots & \ddots & \vdots & \ddots & \ddots & \vdots & \ddots & \vdots \\
0 & \cdots & a_{n1} & \cdots & \cdots & b_{n1} & \cdots & a_{nn}+b_{nn}
\end{bmatrix}.
\end{aligned} \tag{6}$$

The equations above seem overwhelming. Nevertheless, as $\mathbf{\Lambda}$ is a diagonal matrix, $\mathbf{\Lambda} \oplus \mathbf{\Lambda}$ is also a diagonal matrix, and the first element $\mathbf{\Lambda} \oplus \mathbf{\Lambda}$ is the only one approaching zero. This means the term $[-(\mathbf{\Lambda} \oplus \mathbf{\Lambda})]^{-1}$ is a diagonal matrix as well, with only the first element approaching infinity. If the first element of $\mathbf{\Sigma}'$ is not zero, only the first element of $\mathbf{\Sigma}'_\infty$, which is the variance of the first new axis $\text{var}(X_1')$, will approach infinity when the system approaches the bifurcation point.

$$\begin{aligned}
\text{vec}(\mathbf{\Sigma}'_\infty) &= \begin{bmatrix} \text{var}(X_1') \\ \text{cov}(X_1', X_2') \\ \vdots \end{bmatrix} \\
&= \begin{bmatrix} -2\lambda_1 & 0 & \cdots \\ 0 & -\lambda_1-\lambda_2 & \cdots \\ \cdots & \cdots & \cdots \end{bmatrix}^{-1} \begin{bmatrix} \sigma'_{11} \\ \sigma'_{12} \\ \vdots \end{bmatrix} \\
&= \begin{bmatrix} \frac{1}{-2\lambda_1} & 0 & \cdots \\ 0 & \frac{1}{-\lambda_1-\lambda_2} & \cdots \\ \cdots & \cdots & \cdots \end{bmatrix} \begin{bmatrix} \sigma'_{11} \\ \sigma'_{12} \\ \vdots \end{bmatrix} \\
&\to \begin{bmatrix} \infty & 0 & \cdots \\ 0 & \frac{1}{-\lambda_2} & \cdots \\ \cdots & \cdots & \cdots \end{bmatrix} \begin{bmatrix} \sigma'_{11} \\ \sigma'_{12} \\ \vdots \end{bmatrix}.
\end{aligned} \tag{7}$$

The auto-covariance of $\boldsymbol{X}'(t)$ can be calculated with the following formulas.

$$\lim_{t\to\infty} \text{cov}(\boldsymbol{X}'(t), \boldsymbol{X}'(t+\Delta t)) = e^{\mathbf{\Lambda}\Delta t}\mathbf{\Sigma}'_\infty. \tag{8}$$

As $\mathbf{\Lambda}$ is a diagonal matrix, its exponential is a matrix with the exponential of diagonal elements:

$$e^{\mathbf{\Lambda}\Delta t} = \text{diag}(e^{\lambda_1\Delta t}, e^{\lambda_2\Delta t}, ..., e^{\lambda_n\Delta t}), \tag{9}$$

in which the first element approaches 1, and other elements are between 0 and 1. Therefore, the first element of the auto-covariance matrix, which means the auto-covariance of the first new axis $\text{cov}(X_1'(t), X_1'(t+\Delta t))$ will approach $\text{var}(X_1')$.

Finally, we derive the situation back to the original coordinates. Because $\mathbf{\Sigma}_\infty = \boldsymbol{Q}\mathbf{\Sigma}'_\infty\boldsymbol{Q}^T$, the variance and covariance of all the variables with non-zero loading in the first eigenvector will approach infinity, and their correlations will approach 1.

$$\begin{aligned}
\mathbf{\Sigma}_\infty &= \begin{bmatrix} a_1 & b_1 & \cdots \\ a_2 & b_2 & \cdots \\ \cdots & \cdots & \cdots \end{bmatrix} \begin{bmatrix} \text{var}(X_1') & 0 & \cdots \\ 0 & \text{var}(X_2') & \cdots \\ \cdots & \cdots & \cdots \end{bmatrix} \begin{bmatrix} a_1 & a_2 & \cdots \\ b_1 & b_2 & \cdots \\ \cdots & \cdots & \cdots \end{bmatrix} \\
&= \begin{bmatrix} a_1^2\text{var}(X_1')^2 + b_1^2\text{var}(X_2')^2 + \cdots & a_1a_2\text{var}(X_1')^2 + b_1b_2\text{var}(X_2')^2 + \cdots & \cdots \\ a_1a_2\text{var}(X_1')^2 + b_1b_2\text{var}(X_2')^2 + \cdots & a_2^2\text{var}(X_1')^2 + b_2^2\text{var}(X_2')^2 + \cdots & \cdots \\ \cdots & \cdots & \cdots \end{bmatrix} \to \begin{bmatrix} \infty & \infty & \cdots \\ \infty & \infty & \cdots \\ \cdots & \cdots & \cdots \end{bmatrix}.
\end{aligned} \tag{10}$$

$$\lim_{t\to\infty} \operatorname{corr}(X_1, X_2) = \frac{\operatorname{cov}(x_1, x_2)}{\sigma_1 \sigma_2} = \frac{a_1 a_2 \operatorname{var}(X_1')^2 + b_1 b_2 \operatorname{var}(X_2')^2 + \cdots}{\sqrt{a_1^2 \operatorname{var}(X_1')^2 + b_1^2 \operatorname{var}(X_2')^2 + \cdots + a_2^2 \operatorname{var}(X_1')^2 + b_2^2 \operatorname{var}(X_2')^2 + \cdots}} \to 1. \tag{11}$$

The same also holds for auto-correlation.

The eigenvalues and eigenvectors of $\boldsymbol{J}$ may contain complex numbers even if we are working with a real function. This is because the local dynamic around the equilibrium point may not only contain shrinking and growing tendencies but also contain rotating. When there are complex eigenvalues and eigenvectors, they always appear in pairs. The imaginary part of the eigenvalues and eigenvectors of $\boldsymbol{J}$ represents how the system tends to rotate.

If there are complex eigenvalues, we can do a block diagonalization instead of a plain diagonalization (Margalit & Rabinoff, 2019). In a block diagonalization, for each conjugated pair of eigenvalues and eigenvectors, we use the real and the imaginary part of the eigenvectors $[\operatorname{Re}(v)\,\operatorname{Im}(v)]$ as the vectors for coordinates transformation, and we use a 2×2 block $\begin{bmatrix} \operatorname{Re}(\lambda) & \operatorname{Im}(\lambda) \\ -\operatorname{Im}(\lambda) & \operatorname{Re}(\lambda) \end{bmatrix}$ to represent the rotating and shrinking or growing tendencies together.

We assume the first pair of complex eigenvalues are the dominant eigenvalues of $\boldsymbol{J}$, and only the real parts of them approach zero before the bifurcation point. In this case,

$$\begin{aligned}
\operatorname{vec}(\boldsymbol{\Sigma}'_\infty) &= \begin{bmatrix} \operatorname{var}(X_1') \\ \operatorname{cov}(X_1', X_2') \\ \operatorname{cov}(X_1', X_3') \\ \vdots \\ \operatorname{cov}(X_2', X_1') \\ \operatorname{var}(X_2') \\ \operatorname{cov}(X_2', X_3') \\ \vdots \end{bmatrix} \\
&= \begin{bmatrix}
-2\operatorname{Re}(\lambda_1) & -\operatorname{Im}(\lambda_1) & 0 & \cdots & -\operatorname{Im}(\lambda_1) & 0 & 0 & \cdots \\
\operatorname{Im}(\lambda_1) & -2\operatorname{Re}(\lambda_1) & 0 & \cdots & 0 & -\operatorname{Im}(\lambda_1) & 0 & \cdots \\
0 & 0 & -\operatorname{Re}(\lambda_1) - \lambda_3 & \cdots & 0 & 0 & -\operatorname{Im}(\lambda_1) & \cdots \\
\cdots & \cdots & \cdots & \cdots & \cdots & \cdots & \cdots & \cdots \\
\operatorname{Im}(\lambda_1) & 0 & 0 & \cdots & -2\operatorname{Re}(\lambda_1) & -\operatorname{Im}(\lambda_1) & 0 & \cdots \\
0 & \operatorname{Im}(\lambda_1) & 0 & \cdots & -\operatorname{Im}(\lambda_1) & -2\operatorname{Re}(\lambda_1) & 0 & \cdots \\
0 & 0 & \operatorname{Im}(\lambda_1) & \cdots & 0 & 0 & -\operatorname{Re}(\lambda_1) - \lambda_3 & \cdots \\
\cdots & \cdots & \cdots & \cdots & \cdots & \cdots & \cdots & \cdots
\end{bmatrix}^{-1} \begin{bmatrix} \sigma'_{11} \\ \sigma'_{12} \\ \sigma'_{13} \\ \vdots \\ \sigma'_{21} \\ \sigma'_{22} \\ \sigma'_{23} \\ \vdots \end{bmatrix}.
\end{aligned} \tag{12}$$

To simplify the calculation of the inverse, we change the order of the matrix and vectors to make the values that approach infinity all to the upper-left corner of the matrix. The matrix then becomes a block diagonal matrix that we can obtain the invert of each block separately.

$$
\begin{bmatrix} \mathrm{var}(X_1') \\ \mathrm{cov}(X_1', X_2') \\ \mathrm{cov}(X_2', X_1') \\ \mathrm{var}(X_2') \\ \mathrm{cov}(X_1', X_3') \\ \mathrm{cov}(X_2', X_3') \\ \vdots \\ \vdots \end{bmatrix}
$$

$$
= \begin{bmatrix}
-2\,\mathrm{Re}(\lambda_1) & -\mathrm{Im}(\lambda_1) & -\mathrm{Im}(\lambda_1) & 0 & 0 & 0 & \cdots & \cdots \\
\mathrm{Im}(\lambda_1) & -2\,\mathrm{Re}(\lambda_1) & 0 & -\mathrm{Im}(\lambda_1) & 0 & 0 & \cdots & \cdots \\
\mathrm{Im}(\lambda_1) & 0 & -2\,\mathrm{Re}(\lambda_1) & -\mathrm{Im}(\lambda_1) & 0 & 0 & \cdots & \cdots \\
0 & \mathrm{Im}(\lambda_1) & \mathrm{Im}(\lambda_1) & -2\,\mathrm{Re}(\lambda_1) & 0 & 0 & \cdots & \cdots \\
0 & 0 & 0 & 0 & -\mathrm{Re}(\lambda_1)-\lambda_3 & -\mathrm{Im}(\lambda_1) & \cdots & \cdots \\
0 & 0 & 0 & 0 & \mathrm{Im}(\lambda_1) & -\mathrm{Re}(\lambda_1)-\lambda_3 & \cdots & \cdots \\
\cdots & \cdots & \cdots & \cdots & \cdots & \cdots & \cdots & \cdots \\
\cdots & \cdots & \cdots & \cdots & \cdots & \cdots & \cdots & \cdots
\end{bmatrix}^{-1}
\begin{bmatrix} \sigma_{11}' \\ \sigma_{12}' \\ \sigma_{21}' \\ \sigma_{22}' \\ \sigma_{13}' \\ \sigma_{23}' \\ \vdots \\ \vdots \end{bmatrix}, \qquad (13)
$$

$$
= \left[\begin{array}{c|c|c} \boldsymbol{A}_1 & \mathbf{0} & \cdots \\ \hline \mathbf{0} & \boldsymbol{A}_2 & \cdots \\ \hline \cdots & \cdots & \cdots \end{array}\right]
\left[\begin{array}{c} \sigma_{11}' \\ \sigma_{12}' \\ \sigma_{21}' \\ \sigma_{22}' \\ \hline \sigma_{13}' \\ \sigma_{23}' \\ \hline \vdots \end{array}\right]
$$

in which (symbolic calculation performed with Mathematica, Wolfram Research, Inc., 2022)

$$
\begin{aligned}
&\boldsymbol{A}_1 \\
&= \begin{bmatrix}
\frac{-8\,\mathrm{Re}(\lambda_1)^3-4\,\mathrm{Re}(\lambda_1)\,\mathrm{Im}(\lambda_1)^2}{16\,\mathrm{Re}(\lambda_1)^4+16\,\mathrm{Re}(\lambda_1)^2\,\mathrm{Im}(\lambda_1)^2} & \frac{4\,\mathrm{Re}(\lambda_1)^2\,\mathrm{Im}(\lambda_1)}{16\,\mathrm{Re}(\lambda_1)^4+16\,\mathrm{Re}(\lambda_1)^2\,\mathrm{Im}(\lambda_1)^2} & \frac{4\,\mathrm{Re}(\lambda_1)^2\,\mathrm{Im}(\lambda_1)}{16\,\mathrm{Re}(\lambda_1)^4+16\,\mathrm{Re}(\lambda_1)^2\,\mathrm{Im}(\lambda_1)^2} & -\frac{4\,\mathrm{Re}(\lambda_1)\,\mathrm{Im}(\lambda_1)^2}{16\,\mathrm{Re}(\lambda_1)^4+16\,\mathrm{Re}(\lambda_1)^2\,\mathrm{Im}(\lambda_1)^2} \\
-\frac{4\,\mathrm{Re}(\lambda_1)^2\,\mathrm{Im}(\lambda_1)}{16\,\mathrm{Re}(\lambda_1)^4+16\,\mathrm{Re}(\lambda_1)^2\,\mathrm{Im}(\lambda_1)^2} & \frac{-8\,\mathrm{Re}(\lambda_1)^3-4\,\mathrm{Re}(\lambda_1)\,\mathrm{Im}(\lambda_1)^2}{16\,\mathrm{Re}(\lambda_1)^4+16\,\mathrm{Re}(\lambda_1)^2\,\mathrm{Im}(\lambda_1)^2} & \frac{4\,\mathrm{Re}(\lambda_1)\,\mathrm{Im}(\lambda_1)^2}{16\,\mathrm{Re}(\lambda_1)^4+16\,\mathrm{Re}(\lambda_1)^2\,\mathrm{Im}(\lambda_1)^2} & \frac{4\,\mathrm{Re}(\lambda_1)^2\,\mathrm{Im}(\lambda_1)}{16\,\mathrm{Re}(\lambda_1)^4+16\,\mathrm{Re}(\lambda_1)^2\,\mathrm{Im}(\lambda_1)^2} \\
-\frac{4\,\mathrm{Re}(\lambda_1)^2\,\mathrm{Im}(\lambda_1)}{16\,\mathrm{Re}(\lambda_1)^4+16\,\mathrm{Re}(\lambda_1)^2\,\mathrm{Im}(\lambda_1)^2} & \frac{4\,\mathrm{Re}(\lambda_1)\,\mathrm{Im}(\lambda_1)^2}{16\,\mathrm{Re}(\lambda_1)^4+16\,\mathrm{Re}(\lambda_1)^2\,\mathrm{Im}(\lambda_1)^2} & \frac{-8\,\mathrm{Re}(\lambda_1)^3-4\,\mathrm{Re}(\lambda_1)\,\mathrm{Im}(\lambda_1)^2}{16\,\mathrm{Re}(\lambda_1)^4+16\,\mathrm{Re}(\lambda_1)^2\,\mathrm{Im}(\lambda_1)^2} & \frac{4\,\mathrm{Re}(\lambda_1)^2\,\mathrm{Im}(\lambda_1)}{16\,\mathrm{Re}(\lambda_1)^4+16\,\mathrm{Re}(\lambda_1)^2\,\mathrm{Im}(\lambda_1)^2} \\
-\frac{4\,\mathrm{Re}(\lambda_1)\,\mathrm{Im}(\lambda_1)^2}{16\,\mathrm{Re}(\lambda_1)^4+16\,\mathrm{Re}(\lambda_1)^2\,\mathrm{Im}(\lambda_1)^2} & -\frac{4\,\mathrm{Re}(\lambda_1)^2\,\mathrm{Im}(\lambda_1)}{16\,\mathrm{Re}(\lambda_1)^4+16\,\mathrm{Re}(\lambda_1)^2\,\mathrm{Im}(\lambda_1)^2} & -\frac{4\,\mathrm{Re}(\lambda_1)^2\,\mathrm{Im}(\lambda_1)}{16\,\mathrm{Re}(\lambda_1)^4+16\,\mathrm{Re}(\lambda_1)^2\,\mathrm{Im}(\lambda_1)^2} & \frac{-8\,\mathrm{Re}(\lambda_1)^3-4\,\mathrm{Re}(\lambda_1)\,\mathrm{Im}(\lambda_1)^2}{16\,\mathrm{Re}(\lambda_1)^4+16\,\mathrm{Re}(\lambda_1)^2\,\mathrm{Im}(\lambda_1)^2}
\end{bmatrix} \\
&\to \begin{bmatrix}
-\frac{1}{4\,\mathrm{Re}(\lambda_1)} & \frac{1}{4\,\mathrm{Im}(\lambda_1)} & \frac{1}{4\,\mathrm{Im}(\lambda_1)} & -\frac{1}{4\,\mathrm{Re}(\lambda_1)} \\
-\frac{1}{4\,\mathrm{Im}(\lambda_1)} & -\frac{1}{4\,\mathrm{Re}(\lambda_1)} & \frac{1}{4\,\mathrm{Re}(\lambda_1)} & \frac{1}{4\,\mathrm{Im}(\lambda_1)} \\
-\frac{1}{4\,\mathrm{Im}(\lambda_1)} & \frac{1}{4\,\mathrm{Re}(\lambda_1)} & -\frac{1}{4\,\mathrm{Re}(\lambda_1)} & \frac{1}{4\,\mathrm{Im}(\lambda_1)} \\
-\frac{1}{4\,\mathrm{Re}(\lambda_1)} & -\frac{1}{4\,\mathrm{Im}(\lambda_1)} & -\frac{1}{4\,\mathrm{Im}(\lambda_1)} & -\frac{1}{4\,\mathrm{Re}(\lambda_1)}
\end{bmatrix} \\
&\boldsymbol{A}_2 \\
&= \begin{bmatrix}
\frac{-\mathrm{Re}(\lambda_1)-\lambda_3}{\mathrm{Re}(\lambda_1)^2+2\,\mathrm{Re}(\lambda_1)\lambda_3+\mathrm{Im}(\lambda_1)^2+\lambda_3^2} & \frac{\mathrm{Im}(\lambda_1)}{\mathrm{Re}(\lambda_1)^2+2\,\mathrm{Re}(\lambda_1)\lambda_3+\mathrm{Im}(\lambda_1)^2+\lambda_3^2} \\
-\frac{\mathrm{Im}(\lambda_1)}{\mathrm{Re}(\lambda_1)^2+2\,\mathrm{Re}(\lambda_1)\lambda_3+\mathrm{Im}(\lambda_1)^2+\lambda_3^2} & \frac{-\mathrm{Re}(\lambda_1)-\lambda_3}{\mathrm{Re}(\lambda_1)^2+2\,\mathrm{Re}(\lambda_1)\lambda_3+\mathrm{Im}(\lambda_1)^2+\lambda_3^2}
\end{bmatrix} \to \begin{pmatrix} -\frac{1}{\lambda_3} & 0 \\ 0 & -\frac{1}{\lambda_3} \end{pmatrix}.
\end{aligned} \qquad (14)
$$

Because $\sigma_{12}' = \sigma_{21}'$, $\mathrm{cov}(X_1', X_2') = \mathrm{cov}(X_2', X_1') = 0$. As long as σ_{11}' and σ_{22}' are not both zero, $\mathrm{var}(X_1')$ and $\mathrm{var}(X_2')$ will both approach infinity. Because λ_3 does not approach zero, both $\mathrm{cov}(X_1', X_3')$ and $\mathrm{cov}(X_2', X_3')$ will remain finite.

Following the same procedure as used for the real-valued case, it can be proved that the variance of any variables that have non-zero loading in either the real part or the imaginary part of the first eigenvector will approach infinity. Their correlation and auto-correlation will approach 1 as long as the two variables of consideration both belong to either the real part or the imaginary part of the first eigenvector.

To summarize, with the aforementioned assumptions, we can derive the fact that the variables involved in this critical direction should have the variance, (auto)covariance, and (auto)correlations increasing before the transition.

Examples with the bivariate cusp model

Since the derivation above may be difficult to understand, here we provide an example with the bivariate cusp model (Figure 1A in the main text; also described in Supplementary Materials B). Note that this is a special case of the general derivation above.

The model is specified as follows:

$$\begin{aligned} a &= \frac{\sqrt{2}}{2}x + \frac{\sqrt{2}}{2}y, \\ b &= \frac{\sqrt{2}}{2}x - \frac{\sqrt{2}}{2}y, \\ U &= \frac{1}{4}a^4 - \frac{3}{2}a^2 + \lambda a + b^2 \\ &= \frac{1}{16}(x+y)^4 - \frac{3}{4}(x+y)^2 + \frac{\sqrt{2}\lambda}{2}(x+y) + \frac{1}{2}(x-y)^2. \end{aligned} \tag{15}$$

The partial derivatives of U with respect to x and y are as follows:

$$\begin{aligned} \frac{\partial U}{\partial x} &= \frac{1}{4}(x+y)^3 - \frac{3}{2}(x+y) + \frac{\sqrt{2}\lambda}{2} + (x-y), \\ \frac{\partial U}{\partial y} &= \frac{1}{4}(x+y)^3 - \frac{3}{2}(x+y) + \frac{\sqrt{2}\lambda}{2} - (x-y). \end{aligned} \tag{16}$$

The force on the system is the gradient of U, namely $\boldsymbol{F} = \begin{bmatrix} -\frac{\partial U}{\partial x} \\ -\frac{\partial U}{\partial y} \end{bmatrix}$. Therefore, the Jacobian of the system is the following:

$$\begin{aligned} \boldsymbol{J} &= \begin{bmatrix} \frac{\partial F_x}{\partial x} & \frac{\partial F_x}{\partial y} \\ \frac{\partial F_y}{\partial x} & \frac{\partial F_y}{\partial y} \end{bmatrix} \\ &= \begin{bmatrix} -\frac{3}{4}(x+y)^2 + \frac{1}{2} & -\frac{3}{4}(x+y)^2 + \frac{5}{2} \\ -\frac{3}{4}(x+y)^2 + \frac{5}{2} & -\frac{3}{4}(x+y)^2 + \frac{1}{2} \end{bmatrix} \\ &= \begin{bmatrix} -\frac{1}{\sqrt{2}} & \frac{1}{\sqrt{2}} \\ \frac{1}{\sqrt{2}} & \frac{1}{\sqrt{2}} \end{bmatrix} \begin{bmatrix} -2 & 0 \\ 0 & -\frac{3}{2}\left(x^2+2xy+y^2-2\right) \end{bmatrix} \begin{bmatrix} -\frac{1}{\sqrt{2}} & \frac{1}{\sqrt{2}} \\ \frac{1}{\sqrt{2}} & \frac{1}{\sqrt{2}} \end{bmatrix} \end{aligned} \tag{17}$$

At the equilibrium points, both partial derivatives should be zero. Comparing the two equations above, it is obvious that $x - y = 0$, $x = y$. Therefore, at the equilibrium point, we have the following condition:

$$\frac{\partial U}{\partial x} = \frac{\partial U}{\partial y} = 2x^3 - 3x + \frac{\sqrt{2}\lambda}{2} = 0 \tag{18}$$

The closed-form solution for this cubic equation with a parameter has a complex form. Interested readers may use numerical software (e.g., Mathematica, Wolfram Research, Inc., 2022) to observe how the solution differs with different λ values. Here, we only provide the conclusion about the asymptotic behavior close to the bifurcation point. As $\lambda \to 2^-$, the stable equilibrium point at the negative half approaches $(-\frac{\sqrt{2}}{2}, -\frac{\sqrt{2}}{2})$ and the Jacobian approaches $\begin{bmatrix} -1 & 1 \\ 1 & -1 \end{bmatrix}$. The two eigenvalues of the Jacobian then approach 0 and -2 (which can also be seen from the decomposition in Eq. 17).

Following Eq. 5, the variance-covariance matrix of the system under noise is given by the following (note that, as we have the Jacobian, we calculate the variance-covariance matrix in the original coordinates):

$$\begin{aligned} \text{vec}\left(\boldsymbol{\Sigma}_\infty\right) &= \left[-(\boldsymbol{J} \oplus \boldsymbol{J})\right]^{-1} \text{vec}(\boldsymbol{\Sigma}) \\ &\to \begin{bmatrix} 2 & -1 & -1 & 0 \\ -1 & 2 & 0 & -1 \\ -1 & 0 & 2 & -1 \\ 0 & -1 & -1 & 2 \end{bmatrix}^{-1} \text{vec}(\boldsymbol{\Sigma}), \end{aligned} \tag{19}$$

in which the first matrix is singular. To know what exact elements of the matrix are approaching infinity, we do a calculation when λ is a little smaller than 2, that the equilibrium is a little to the negative side of $(-\frac{\sqrt{2}}{2}, -\frac{\sqrt{2}}{2})$. Assume the Jacobian is now $\begin{bmatrix} -1.01 & 1.01 \\ 1.01 & -1.01 \end{bmatrix}$, we have (numerical calculation was conducted by Mathematica, Wolfram Research, Inc., 2022):

$$
\begin{aligned}
\operatorname{vec}\left(\boldsymbol{\Sigma}_{\infty}\right) &= [-(\boldsymbol{J} \oplus \boldsymbol{J})]^{-1} \operatorname{vec}(\boldsymbol{\Sigma}) \\
&= \begin{bmatrix} 2.02 & -1.01 & -1.01 & 0 \\ -1.01 & 2.02 & 0 & -1.01 \\ -1.01 & 0 & 2.02 & -1.01 \\ 0 & -1.01 & -1.01 & 2.02 \end{bmatrix}^{-1} \operatorname{vec}(\boldsymbol{\Sigma}) \\
&= \begin{bmatrix} 6.68851 \times 10^{15} & 6.68851 \times 10^{15} & 6.68851 \times 10^{15} & 6.68851 \times 10^{15} \\ 6.68851 \times 10^{15} & 6.68851 \times 10^{15} & 6.68851 \times 10^{15} & 6.68851 \times 10^{15} \\ 6.68851 \times 10^{15} & 6.68851 \times 10^{15} & 6.68851 \times 10^{15} & 6.68851 \times 10^{15} \\ 6.68851 \times 10^{15} & 6.68851 \times 10^{15} & 6.68851 \times 10^{15} & 6.68851 \times 10^{15} \end{bmatrix} \operatorname{vec}(\boldsymbol{\Sigma})
\end{aligned} \tag{20}
$$

Therefore, as long as there is some noise in the system (i.e., $\boldsymbol{\Sigma} \neq \mathbf{0}$), the variance and covariance of x and y will approach infinity.

We also do the same calculation for the second model in the main text (Figure 1B in the main text, also described in Supplementary Materials B). This time the model is specified as:

$$
U = \frac{1}{4}x^4 - \frac{3}{2}x^2 + \lambda x + y^2. \tag{21}
$$

We then have:

$$
\begin{aligned}
\frac{\partial U}{\partial x} &= x^3 - 3x + \lambda, \\
\frac{\partial U}{\partial y} &= 2y.
\end{aligned} \tag{22}
$$

$$
\begin{aligned}
\boldsymbol{J} &= \begin{bmatrix} \frac{\partial F_x}{\partial x} & \frac{\partial F_x}{\partial y} \\ \frac{\partial F_y}{\partial x} & \frac{\partial F_y}{\partial y} \end{bmatrix} \\
&= \begin{bmatrix} -3x^2 + 3 & 0 \\ 0 & -2 \end{bmatrix}
\end{aligned} \tag{23}
$$

When $\lambda \to 2$, the equilibrium point at the negative half approaches $(-1, 0)$, and the Jacobian approaches $\begin{bmatrix} 0 & 0 \\ 0 & -2 \end{bmatrix}$. Again, we consider the case when λ is a little smaller than 2, and $\boldsymbol{J} = \begin{bmatrix} -0.01 & 0 \\ 0 & -2 \end{bmatrix}$. Similarly, we have (numerical calculation was conducted by Mathematica, Wolfram Research, Inc., 2022):

$$
\begin{aligned}
\operatorname{vec}\left(\boldsymbol{\Sigma}_{\infty}\right) &= [-(\boldsymbol{J} \oplus \boldsymbol{J})]^{-1} \operatorname{vec}(\boldsymbol{\Sigma}) \\
&= \begin{bmatrix} 0.02 & 0 & 0 & 0 \\ 0 & 2.01 & 0 & 0 \\ 0 & 0 & 2.01 & 0 \\ 0 & 0 & 0 & 4 \end{bmatrix}^{-1} \operatorname{vec}(\boldsymbol{\Sigma}) \\
&= \begin{bmatrix} 50 & 0 & 0 & 0 \\ 0 & 0.497512 & 0 & 0 \\ 0 & 0 & 0.497512 & 0 \\ 0 & 0 & 0 & 0.25 \end{bmatrix} \operatorname{vec}(\boldsymbol{\Sigma}).
\end{aligned} \tag{24}
$$

Here, as long as there is some noise for x (i.e., $\boldsymbol{\Sigma}_{xx} \neq 0$), the variance of x and x only will approach infinity. Other elements of the variance-covariance matrix will remain small.

References

Dablander, F., Pichler, A., Cika, A., & Bacilieri, A. (2022). Anticipating critical transitions in psychological systems using early warning signals: Theoretical and practical considerations. *Psychological Methods*. https://doi.org/10.1037/met0000450

Margalit, D., & Rabinoff, J. (2019). Eigenvalues and eigenvectors. In *Interactive Linear Algebra*. Retrieved April 19, 2023, from https://textbooks.math.gatech.edu/ila/chap-eigenvalues.html

Meucci, A. (2009, May 14). Review of Statistical Arbitrage, Cointegration, and Multivariate Ornstein-Uhlenbeck. https://doi.org/10.2139/ssrn.1404905

Scheffer, M., Bascompte, J., Brock, W. A., Brovkin, V., Carpenter, S. R., Dakos, V., Held, H., van Nes, E. H., Rietkerk, M., & Sugihara, G. (2009). Early-warning signals for critical transitions. *Nature*, *461*(7260), 53–59. https://doi.org/10.1038/nature08227

Vatiwutipong, P., & Phewchean, N. (2019). Alternative way to derive the distribution of the multivariate Ornstein–Uhlenbeck process. *Advances in Difference Equations*, *2019*(1), 276. https://doi.org/10.1186/s13662-019-2214-1

Wolfram Research, Inc. (2022). Mathematica, Version 13.2. https://www.wolfram.com/mathematica
