# Supplementary material for: Examining the research methods of early warning signals in clinical psychology through a theoretical lens
Source: BMC Psychiatry. 2025 Mar 19;25:261. doi: 10.1186/s12888-025-06688-5 (PMC11924765; doi:10.1186/s12888-025-06688-5)
Supplement: Supplementary file 2 — Supplementary Material 2 [file 12888_2025_6688_MOESM2_ESM.pdf]

Supplementary Materials B: Landscape Illustrations and Simulations

For the manuscript “Examining the Research Methods of Early Warning Signals in Clinical Psychology through a Theoretical Lens.”

Jingmeng Cui, Merlijn Olthof, Fred Hasselman, Anna Lichtwarck-Aschoff

Multivariate landscapes and simulations

In this section, we explain the details of the results shown in Figure 1-3 in the main text. To illustrate different scenarios for landscape changes, we created a set of landscape functions, all of which are based on the general bistable landscape function used by Shi et al. (2016), $U = \frac{1}{4}x^4 - \frac{3}{2}x^2 + \lambda x$. We performed coordinate transformations or added additional components to transform the landscape into certain geometric shapes. Specifications of those landscapes are listed as follows.

System (a) is a coordinate transformation of System (b) (see below) to make both *x*- and *y*-axes involved in the transition:

$$a = \frac{\sqrt{2}}{2}x + \frac{\sqrt{2}}{2}y,$$

$$b = \frac{\sqrt{2}}{2}x - \frac{\sqrt{2}}{2}y,$$

$$U = \frac{1}{4}a^4 - \frac{3}{2}a^2 + \lambda a + b^2.$$

System (b) is a multivariate extension of the original one-dimensional landscape function. It adds the *y*-axis, which takes a quadratic form and does not introduce additional basins:

$$U = \frac{1}{4}x^4 - \frac{3}{2}x^2 + \lambda x + y^2.$$

System (c) added a term, $-2\mathrm{ReLU}(x)y$, to System (b) to show an example in which the starting direction of the transition does not directly point to the endpoint of the transition:

$$U = \frac{1}{4}x^4 - \frac{3}{2}x^2 + \lambda x + y^2 - 2\mathrm{ReLU}(x)y, \text{in which } \mathrm{ReLU}(x) = \max(x, 0).$$

System (d) is a coordinate transformation of System (b) to make the transition along a curve instead of a straight line:

$$\theta = \arctan\frac{y}{x},$$

$$a = \frac{4\theta}{\pi} + 0.5,$$

$$b = 3\left(\sqrt{x^2 + y^2} - 3\right),$$

$$U = \frac{1}{4}a^4 - \frac{3}{2}a^2 + \lambda a + b^2.$$

System (e) is a coordinate transformation of System (b) to make the new attractor a circle instead of a point:

$$a = 3\left(\sqrt{x^2 + y^2}\right) - 1,$$

$$U = \frac{1}{4}a^4 - \frac{3}{2}a^2 + \lambda a.$$

We then draw all the landscape functions in Figure 1. All the examples shown in Figure 1 of the main text are cusp bifurcations.

Taking the gradients from the landscape functions, and adding the stochastic term, we can have the stochastic dynamic equations of the system,

$$\mathrm{d}x = -\frac{\partial U}{\partial x}\mathrm{d}t + \sigma_x \mathrm{d}W_x,$$

$$\mathrm{d}y = -\frac{\partial U}{\partial y}\mathrm{d}t + \sigma_y \mathrm{d}W_y,$$

which can be used to simulate the system with the Euler–Maruyama method. In our simulations, we let the control parameter λ change according to the formula $\lambda = (3 - t)/100$, and we used a timestep of 0.01, a simulation length of 700 time units, and a noise level of $\sigma = 0.3$.

EWS simulation shown in the main text

In this section, we explain the details of the results shown in Figure 4 in the main text.

Again, we use the simple gradient system with noise by Shi et al. (2016) as the model for our simulation. The model contains one state variable, x, and a control parameter, λ. The potential function of the system, U, is specified as follows. Note that this function only differs in a constant coefficient compared with the function introduced in the previous section. This is to make the change rate in the simulations more realistic.

$$U(x, \lambda) = 100\left(\frac{1}{4}x^4 - \frac{3}{2}x^2 + \lambda x\right). \tag{1}$$

The dynamic functions of the system are then specified as

$$\frac{\mathrm{d}x}{\mathrm{d}t} = -\frac{\partial U(x, \lambda)}{\partial x} + \sqrt{2\sigma}\xi(t), \tag{2}$$

where $\mathrm{d}x/\mathrm{d}t$ represents the change rate of x, $\partial U(x, \lambda)/\partial x$ represents the gradient of the potential function with respect to x, σ represents the strength of the noise and was set as 10 in this study (as in Shi et al., 2016), and $\xi(t)$ represents standard white noise. The potential landscapes of the system with different λ, as well as the equilibrium points of the system where $\partial U(x, \lambda)/\partial x = 0$, are shown in Figure B1. For simulating the change of the landscape, the initial value of λ is set as -3, and the changing rate of λ is set as $\mathrm{d}\lambda/\mathrm{d}t = 1$. When the simulation starts, there is only one basin for the system. We refer to this as the *positive phase* because it is in the positive semi-axis of x. As λ increases to -2, the second basin appears, and its stability increases as λ further increases. We refer to this basin as the *negative phase*. When λ increases to

2, the system reaches its bifurcation point. The positive phase of the system disappears and the negative basin becomes the only possible basin. All simulations were numerically performed using the Euler-Maruyama method, with 10^{-4} as the step size and 6 as the total time length. The raw simulation data were subsampled by a factor of 10 to reduce the length of the data. Therefore, the time interval between adjunct time points in the output is 10^{-3}.

Here we also show two early warning indicators that are commonly used in previous empirical studies: increasing variance and increasing autocorrelation function (ACF). All the two parameters were estimated with the overlapping moving window approach. The window size was selected as 200 time points ($\Delta t = 0.2$), and each time the window moved forward for 20 time points ($\Delta t = 0.02$). Here the number of time points in each window is much more than the typical value in empirical studies. We chose this large value because the main purpose of this simulation is to qualitatively show the phenomenon of EWSs, not to provide guidance on the window size for empirical studies. A rather large window size can ensure the stability of the results. Within each window, the variance and lag-1 ACF were calculated. Specifically, the data were linear-detrended within each window before calculating the autocorrelation coefficient. The right-aligned windows were used, which means that, for example, the variance calculated within the window from $t = 0$ to $t = 0.2$ is regarded as the variance at $t = 0.2$. Thus, no future information is included in the moving windows statistics.

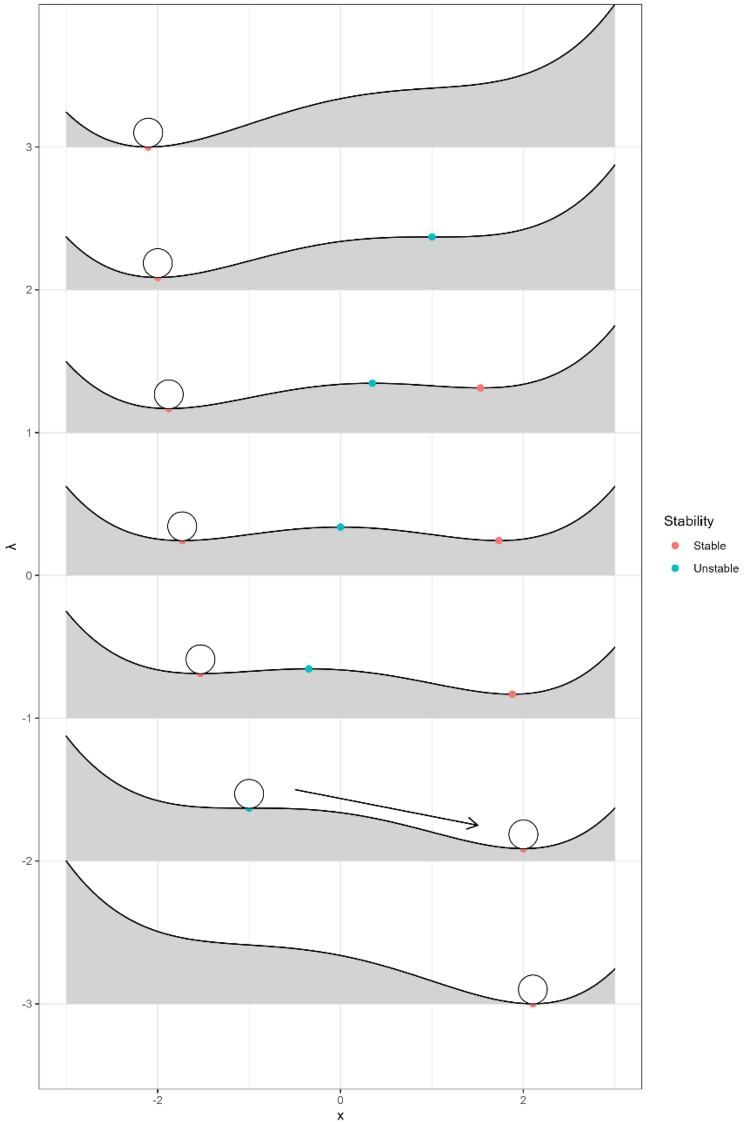

Figure B1. Illustration of a cusp bifurcation specified by Equations 1. The gray areas represent the potential landscapes for different λ values. The red dots represent the stable equilibrium points and the blue points represent the unstable equilibrium points. The white circles represent how the system experiences a sudden change during a cusp bifurcation. At the bifurcation point ($\lambda = 2$), the state of the system became unstable, so it transitions to another state.

Additional simulation results

In this section, we provide some additional simulation results related to, but not reported in the main text.

Consequences of calculating EWSs not strictly before the transition. If the EWS calculation window includes the transition itself, the increase in variance and autocorrelation may be falsely taken as evidence of EWSs. To illustrate this point, we conducted simulations for another condition, in which the transition is purely driven by a large fluctuation so that there are no EWSs before the transition. This condition is set up the same as described in the *EWS simulation shown in the main text*, except that the parameter λ is held constant at 0, which means that the potential function U does not change through the simulation and that there is a strong noise $\Delta x = -3$ at $t = 3$ that pushes the system to the negative phase. We use the time that the system first crosses the barrier as the time of the transition (t_{trans}), and we use Kendall's τ calculated with the Kendall package (McLeod, 2011) to evaluate the trends of variance and autocorrelation. We investigated three types of ranges in the current research: (1) strictly before the transition, for which τ was calculated in the range from $t_{\text{trans}} - 1.5$ to t_{trans}; (2) roughly before the transition, for which τ was calculated in the range from $t_{\text{trans}} - 1.5$ to $t_{\text{trans}} + 0.5$; and (3) around the transition, for which τ was calculated in the range from $t_{\text{trans}} - 1.5$ to $t_{\text{trans}} + 1.5$. These conditions were set to mimic different empirical studies where EWSs are calculated strictly before the transition (when the transition indicator is calculated in at least the same frequency as EWSs), roughly before the transition (when the transition indicator is calculated through the whole period but in a lower frequency as EWSs), and in a large range that may contain a transition (when the transition indicator is only calculated before or after the whole study period). The range sizes are set as roughly one order of magnitude larger than the

window sizes for moving window statistics, which is often the case in empirical studies. For each condition, the simulation was replicated 10^3 times and the results and statistical indicators were recorded. Examples of the simulated time series are shown in Figure B2, and the distributions of τ in repeated simulations are shown in Figure B3. From the simulation results, it is clear that if the EWS calculation window is not strictly before the transition, the statistical effect of the transition itself may be falsely taken as evidence of EWSs, and true EWSs may not be detected because the decrease in variance and ACF may average out the true EWSs.

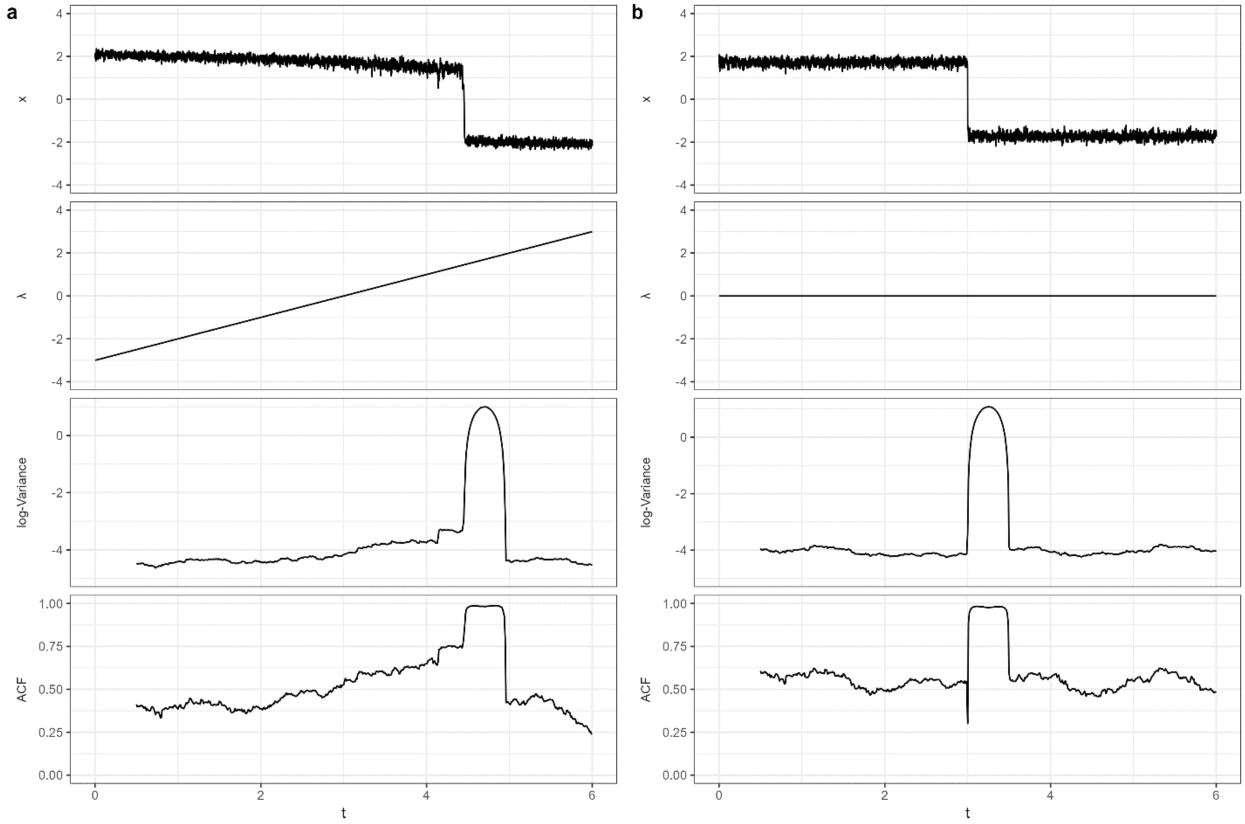

Figure B2. Simulation examples for sudden changes caused by (a) the bifurcation of the system, and (2) a large fluctuation

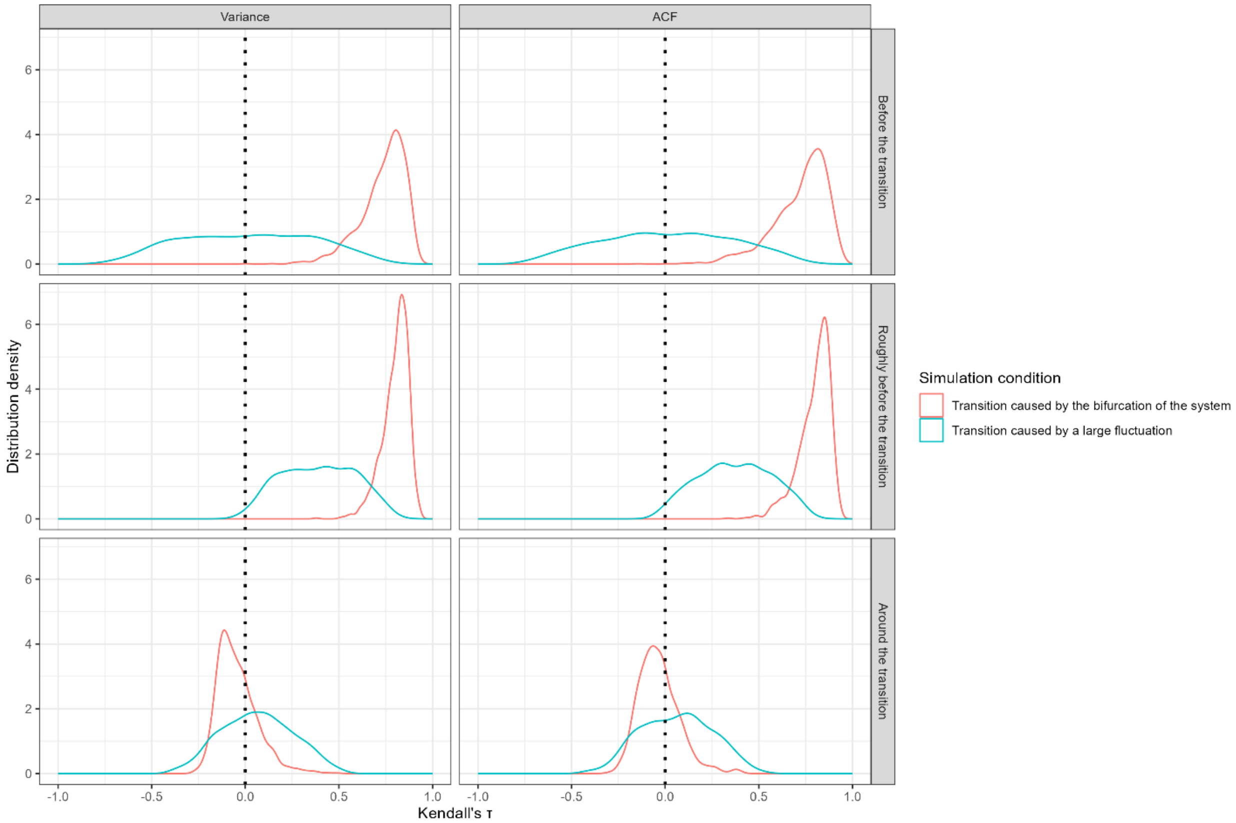

Figure B3. Trends of variance and ACF represented with the distribution of Kendall's τ from 10^3 simulations, for two simulation conditions and different periods.

Consequences of using inconsistent variables for calculating EWSs and detecting sudden changes. In the main text, we explained why, from theoretical considerations, researchers should use a consistent set of variables to calculate EWSs and detect sudden changes. Here, we use a simplified simulation example to illustrate the possible consequences of using different variables in empirical studies, with some variables *only* used for calculating EWSs and some *only* used for detecting sudden changes. The model we used here is very similar to the one used in the section *Consequences of calculating EWSs not strictly before the transition*, but there are two sets of equations for two variables x_1 andx_2:

$$V_1(x_1, \lambda_1) = 100\left(\frac{1}{4}x_1^4 - \frac{3}{2}x_1^2 + \lambda_1 x_1\right), \tag{3}$$

$$\frac{\mathrm{d}x_1}{\mathrm{d}t} = -\frac{\partial V_1(x_1, \lambda_1)}{\partial x_1} + \sqrt{2\sigma_1}\xi_1(t), \tag{4}$$

$$V_2(x_2, \lambda_2) = 100\left(\frac{1}{4}x_2^4 - \frac{3}{2}x_2^2 + \lambda_2 x_2\right), \tag{5}$$

$$\frac{\mathrm{d}x_2}{\mathrm{d}t} = -\frac{\partial V_2(x_2, \lambda_2)}{\partial x_2} + \sqrt{2\sigma_2}\xi_2(t). \tag{6}$$

We set $\sigma_1 = 400$ and $\sigma_2 = 10$ so that x_1 is influenced by strong noise and does not show a single, clear transition, and x_2 has a transition when λ_2 approaches 2 (as in Shi et al., 2016). We further include a weak relationship between x_1 and x_2 by associating λ_1 and λ_2. The relationship of λ_1 and λ_2 can, in principle, take any form. We illustrate two simple conditions: (a) $\lambda_2 = \lambda_1$, (b) $\lambda_2 = \lambda_1 + 2$. The starting value and changing rate of λ_1 were set the same as in section *Consequences of calculating EWSs not strictly before the transition*: the initial value of λ_1 is -3 and $\mathrm{d}\lambda_1/\mathrm{d}t = 1$. The simulation methods (e.g., simulation length, step size, etc.) are the same as in the section *Consequences of calculating EWSs not strictly before the transition*. Again, we show simulation examples in Figure B4, and the distribution of Kendall's τ in repeated simulations in Figure B5. From the simulation results, we can see that if EWSs and sudden changes are detected from different variables, the trend of variance or ACF may not be related to the sudden change.

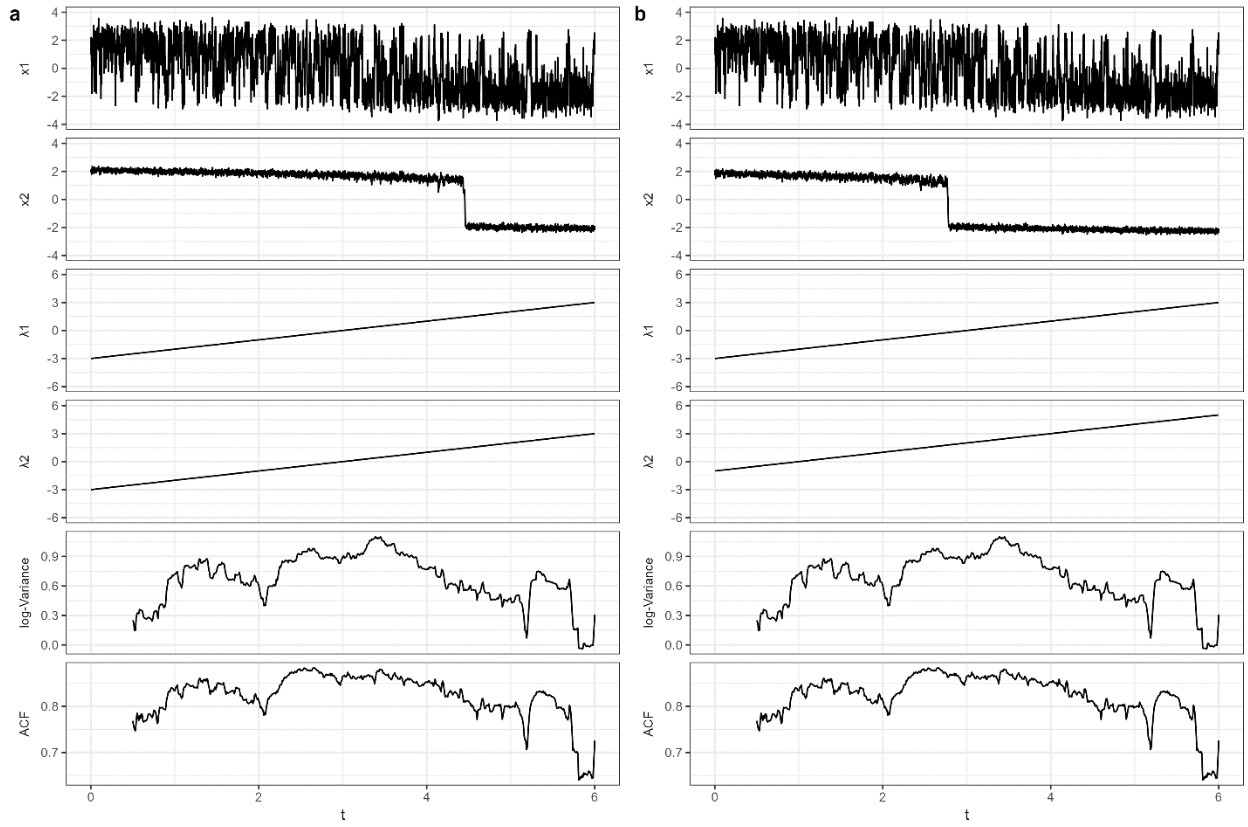

Figure B4. Simulation examples for two conditions.

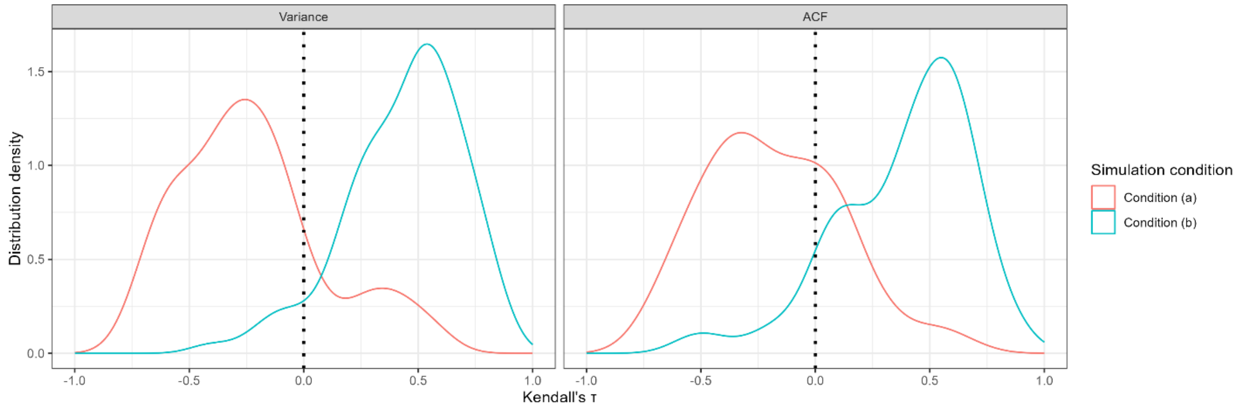

Figure B5. Trends of variance and ACF represented with the distribution of Kendall's τ from 10^3 simulations, for two conditions and different periods.

Code availability.

All the code necessary to replicate the results shown in this file can be found in the OSF repository of this project https://osf.io/f659u/.
